# Gastrointestinal adverse reactions and metabolism–nutrition disorders associated with hypomethylating agents: a pharmacovigilance study with exploratory mechanistic analysis

**DOI:** 10.3389/fnut.2026.1865637

**Published:** 2026-06-30

**Authors:** Mei Xiang, Junjun Li, Xingxing Long, Cong Luo, Jiaqi Zhu, Zhen Liu, Yixiong Cao, Zeyu Luo, Feng Wen

**Affiliations:** Department of Hematology, The First Affiliated Hospital, Hengyang Medical School, University of South China, Hengyang, Hunan, China

**Keywords:** gastrointestinal toxicity, hypomethylating agents, iron overload, metabolism and nutrition disorders, pharmacovigilance, tumor lysis syndrome

## Abstract

**Background:**

Hypomethylating agents, particularly azacitidine and decitabine, are widely used for the treatment of myelodysplastic syndromes and acute myeloid leukemia. Gastrointestinal adverse events and metabolism- and nutrition-related disorders may compromise nutritional intake, metabolic homeostasis, treatment tolerance, and clinical outcomes. However, real-world evidence regarding these safety profiles remains limited.

**Objective:**

This study aimed to characterize gastrointestinal and metabolism- and nutrition-related adverse event reporting signals associated with azacitidine and decitabine, examine cross-database signal consistency, assess exploratory time-to-onset patterns, and explore potential mechanistic clues.

**Methods:**

FAERS reports from database inception through 2025 Q4 were analyzed for azacitidine and decitabine recorded as suspected drugs. Disproportionality was assessed at the preferred-term level using reporting odds ratio, proportional reporting ratio, Bayesian confidence propagation neural network, and multi-item gamma Poisson shrinker methods. Selected signals were examined in the Canada Vigilance database for cross-database signal consistency. Exploratory time-to-onset patterns were evaluated using Weibull modeling among reports with valid date information, and network toxicology was conducted as a hypothesis-generating analysis.

**Results:**

Fourteen thousand two hundred sixteen azacitidine-related reports and 3,591 decitabine-related reports were included. Neutropenic colitis showed the strongest gastrointestinal disproportionality signal for azacitidine and decitabine, with reporting odds ratios of 23.65 and 26.05, respectively. Tumor lysis syndrome was the most prominent metabolism- and nutrition-related signal, with 165 azacitidine-related reports and 47 decitabine-related reports. Both signals showed consistent disproportional reporting patterns in the Canada Vigilance database. Additional signals included iron overload, hypoalbuminemia, electrolyte disturbances, cachexia, failure to thrive, and decreased appetite. Time-to-onset analysis suggested an early reporting tendency among reports with valid date information. Exploratory network toxicology identified 45 common targets, including *MMP9, PTGS2, CASP3, GSK3B*, and *ADAM17*, suggesting potential involvement of inflammation, apoptosis, matrix remodeling, nitrogen metabolism, folate biosynthesis, lipid metabolism, and insulin resistance pathways.

**Conclusion:**

Azacitidine and decitabine showed clinically relevant gastrointestinal, metabolic, and nutrition-related disproportionality reporting signals in spontaneous reporting databases. Early monitoring of gastrointestinal symptoms, electrolyte balance, tumor lysis indicators, albumin levels, appetite, and iron metabolism may support nutritional risk management and individualized supportive care in patients receiving hypomethylating agents.

## 1 Introduction

Aberrant DNA methylation is an important epigenetic mechanism underlying the initiation and progression of myeloid malignancies, including myelodysplastic syndromes (MDS) and acute myeloid leukemia (AML) (1, 2). Hypermethylation of CpG islands in the promoter regions of tumor suppressor genes can induce gene silencing, thereby promoting clonal proliferation and impaired differentiation of myeloid cells (3). As DNA methyltransferase (DNMT) inhibitors and hypomethylating agents, azacitidine and decitabine were approved by the U.S. Food and Drug Administration (FDA) in 2004 and 2006, respectively, for the treatment of MDS. According to the French-American-British (FAB) classification, their approved indications also include chronic myelomonocytic leukemia (CMML) (4, 5). Subsequently, venetoclax in combination with azacitidine or decitabine received accelerated FDA approval in 2018 and regular approval in 2020 for the treatment of newly diagnosed AML in adults aged ≥75 years or in those who are ineligible for intensive induction chemotherapy because of comorbidities (6). In recent years, combination regimens based on hypomethylating agents, such as those combined with venetoclax, immune checkpoint inhibitors, or targeted therapies, have been widely used in clinical practice and have substantially improved survival outcomes in patients with high-risk MDS and older patients with AML. However, these developments have also markedly changed the exposed population and patterns of drug use.

Although the clinical efficacy of azacitidine and decitabine has been confirmed in multiple randomized controlled trials, the management of treatment-related adverse events remains a major challenge in clinical practice. Recent evidence from antineoplastic treatment safety studies has further emphasized that adverse event profiles and treatment discontinuation due to toxicity are important considerations in therapeutic decision-making (7). According to the FDA-approved prescribing information, the most common adverse reactions associated with these two agents include myelosuppression, nausea, vomiting, diarrhea, and constipation. Gastrointestinal toxicity not only directly affects patients' quality of life and nutritional intake but may also contribute to serious complications, such as infection and bleeding, secondary to severe myelosuppression (4, 8). In addition, metabolism and nutrition disorders, including electrolyte imbalance, hypoalbuminemia, and tumor lysis syndrome, should not be overlooked during treatment with hypomethylating agents. These abnormalities may arise not only from the cytotoxic effects of the drugs themselves and their interference with pyrimidine metabolism, but also from interactions among multiple factors, including tumor burden, transfusion dependence, and patients' baseline nutritional status (9). Notably, with the approval of new formulations such as oral azacitidine (CC-486) and oral decitabine combined with cedazuridine (INQOVI), the accessibility of hypomethylating agents and the size of the exposed population have further expanded, creating an urgent clinical need for a more detailed characterization of their safety profiles (10, 11).

However, current knowledge regarding adverse reactions associated with hypomethylating agents is mainly derived from registration clinical trials. Such trials usually apply strict inclusion and exclusion criteria, have limited representativeness for older patients with comorbidities, and involve relatively short follow-up periods. In contrast, real-world data can reflect drug safety characteristics in broader patient populations and serve as an important complement to clinical trial evidence. The FDA Adverse Event Reporting System (FAERS), one of the largest spontaneous reporting pharmacovigilance databases worldwide, contains adverse event reports submitted by healthcare professionals, pharmaceutical companies, and patients, thereby providing a valuable data resource for the systematic assessment of drug-related adverse reaction signals (12). Although FAERS-based pharmacovigilance studies have been widely conducted for various antineoplastic agents, safety findings from other drug classes cannot be directly extrapolated to hypomethylating agents. Azacitidine and decitabine are mainly used in patients with myelodysplastic syndromes and acute myeloid leukemia, who are often older, cytopenic, nutritionally vulnerable, transfusion-dependent, and increasingly exposed to venetoclax-based combination regimens. In this clinical context, gastrointestinal adverse reactions and metabolism- and nutrition-related disorders are not merely non-specific supportive-care events, but may directly affect infection risk, metabolic homeostasis, nutritional deterioration, treatment continuity, and clinical outcomes. Nevertheless, large-scale real-world studies with external validation specifically focusing on these clinically interconnected toxicity domains in hypomethylating agents remain scarce. Moreover, previous pharmacovigilance studies have often focused on signal detection alone, whereas the temporal characteristics and potential biological mechanisms of these adverse reactions remain insufficiently explored.

Therefore, this study aimed to systematically characterize gastrointestinal adverse reactions and metabolism- and nutrition-related disorders associated with azacitidine and decitabine using real-world pharmacovigilance data. Key safety signals were further examined using an independent external database to strengthen the reliability of the findings. In addition, we evaluated the time-to-onset patterns of these adverse events to identify clinically relevant monitoring windows and explored potential toxicological mechanisms through integration of drug targets, protein-protein interactions, and pathway enrichment analyses. The specific novelty of this study lies in its HMA-centered multidimensional design, which integrates real-world safety assessment, external validation, temporal characterization, and mechanistic exploration to delineate class-relevant and drug-specific gastrointestinal and metabolic/nutritional safety profiles of azacitidine and decitabine. This study may provide pharmacovigilance evidence and mechanistic clues for improving clinical risk management, developing individualized monitoring strategies, and guiding subsequent mechanistic studies of hypomethylating agents.

## 2 Materials and methods

### 2.1 Data source

The adverse drug event (ADE) data used in this study were obtained from the FAERS database, which has been publicly accessible since 2004 and collects adverse event reports from multiple sources, including healthcare professionals, pharmaceutical manufacturers, and patients. To investigate ADEs associated with the hypomethylating agents azacitidine and decitabine, FAERS data from database inception to the fourth quarter of 2025 (Q4 2025) were extracted. The data were subsequently imported into MySQL 15.0 and processed using Navicat Premium 15 to facilitate comprehensive analysis.

### 2.2 Data extraction and analysis

**Figure 1:**
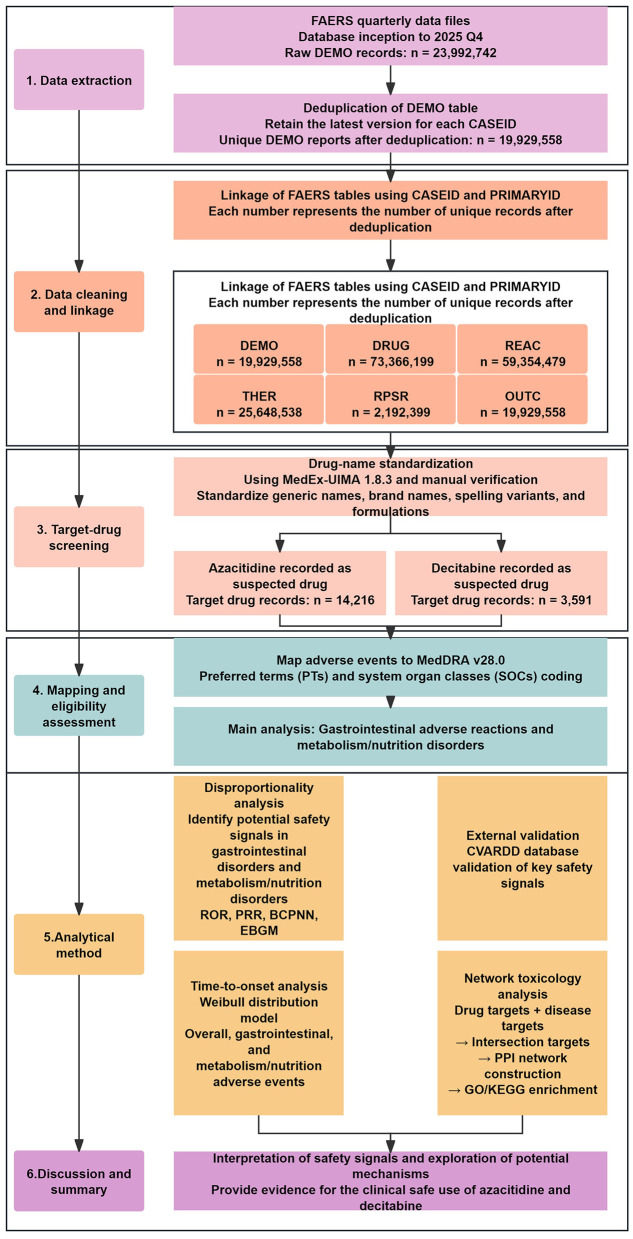
Flowchart of FAERS data selection, preprocessing, and analytical workflow. FAERS data from database inception to 2025 Q4 were extracted, deduplicated, linked across core tables, standardized by drug name, mapped to MedDRA v28.0 terms, and screened for azacitidine- and decitabine-related reports. The final target-drug records included 14,216 azacitidine-related reports and 3,591 decitabine-related reports, which were used for signal detection, external validation, time-to-onset analysis, and network toxicology analysis. FAERS, FDA Adverse Event Reporting System; MedDRA, Medical Dictionary for Regulatory Activities; PT, preferred term; SOC, system organ class; CVARDD, Canada Vigilance Adverse Reaction Online Database; PPI, protein–protein interaction; GO, Gene Ontology; KEGG, Kyoto Encyclopedia of Genes and Genomes.

Azacitidine and decitabine were defined as the target hypomethylating agents in this study. The FAERS data selection and preprocessing workflow is presented in Figure 1. Briefly, FAERS quarterly data files from database inception to the fourth quarter of 2025 were downloaded and imported into SAS and MySQL for data cleaning and management. The DEMO, DRUG, REAC, INDI, THER, RPSR, and OUTC tables were linked using CASEID and PRIMARYID to construct report-level datasets containing demographic information, drug exposure, adverse event terms, indications, and treatment dates where available.

Duplicate reports were removed before analysis. When multiple records shared the same CASEID, the most recent report version was retained according to the reporting date and case version information. Drug names were standardized using MedEx-UIMA 1.8.3, followed by manual verification of generic names, brand names, spelling variants, and formulation-related terms. Reports were then screened to identify cases in which azacitidine or decitabine was recorded as a suspected drug. Reports lacking essential information on the target drug or reported adverse event were excluded from signal analysis. In contrast, reports with missing demographic variables, such as age, sex, or body weight, were not excluded; these variables were summarized as “missing” in the baseline characteristics to avoid unnecessary loss of pharmacovigilance information.

Adverse events were coded using the Medical Dictionary for Regulatory Activities (MedDRA v28.0), and preferred terms (PTs) were mapped to their corresponding system organ classes (SOCs). To characterize clinically relevant organ-system involvement, PTs were further classified according to their primary SOCs. SOCs directly reflecting organ system diseases or clinical symptoms were retained, including infections and infestations, gastrointestinal disorders, nervous system disorders, respiratory disorders, skin and subcutaneous tissue disorders, blood and lymphatic system disorders, cardiac and vascular disorders, musculoskeletal and connective tissue disorders, renal and urinary disorders, metabolism and nutrition disorders, psychiatric disorders, hepatobiliary disorders, eye disorders, immune system disorders, reproductive system and breast disorders, endocrine disorders, and ear and labyrinth disorders. The SOC “General disorders and administration site conditions” was also retained because it reflects systemic symptoms and administration-site reactions.

Clinical characteristics were extracted for descriptive analysis, including sex, age, body weight, reporting country, indication, and reporting year. Underreporting was not treated as an exclusion step because it cannot be identified at the individual-report level in a spontaneous reporting system. Instead, its potential influence on signal interpretation was addressed in the Section 5.

### 2.3 Data mining algorithms

To identify potential associations between hypomethylating agents and reported ADEs, disproportionality analysis was performed at the preferred-term level. For each drug–ADE pair, a 2 × 2 contingency table was constructed by comparing the reporting frequency of a specific ADE for the target drug with the reporting frequency of the same ADE for all other drugs in the FAERS database. In this study, azacitidine and decitabine were analyzed separately as target drugs, and all other drugs in FAERS were used as the reference group.

Four commonly used disproportionality methods were applied for signal detection: the reporting odds ratio (ROR) (13), proportional reporting ratio (PRR) (14), Bayesian confidence propagation neural network (BCPNN) (15), and multi-item gamma Poisson shrinker (MGPS) (16). For the MGPS method, the empirical Bayes geometric mean (EBGM) and its lower confidence limit were used to evaluate signal strength. The formulas and threshold criteria for the four methods are provided in Supplementary Table S1. Briefly, a positive signal was defined as follows: for ROR, the lower limit of the 95% confidence interval was greater than 1 with at least three reports; for PRR, PRR ≥ 2, χ^2^ ≥ 4, and at least three reports; for BCPNN, the lower limit of the 95% credibility interval of the information component was greater than 0; and for MGPS, the lower confidence limit of EBGM exceeded the predefined threshold.

A drug–ADE pair was considered a detected signal if it met the threshold of at least one of the four disproportionality methods. Signals supported by multiple methods were considered more stable and were prioritized for clinical interpretation. Signal strength was mainly described using the ROR value and its 95% confidence interval, while the results of PRR, BCPNN, and MGPS were used as complementary evidence. All statistical analyses were performed using R software (version 4.3.0; R Foundation for Statistical Computing, Vienna, Austria).

It should be noted that disproportionality analysis reflects relative reporting frequency rather than true incidence or absolute risk. Therefore, higher disproportionality estimates indicate stronger reporting associations between the target drug and the ADE, but they do not by themselves establish causality.

### 2.4 Time-to-onset analysis

Time to onset (TTO) was calculated only for reports with sufficient date information. The treatment start date was defined as the start date of azacitidine or decitabine recorded in the THER table, and the adverse event onset date was defined as the event date recorded in the DEMO or REAC-related reporting fields where available. TTO was calculated as the interval between the treatment start date and the adverse event onset date. Reports were included in the TTO analysis only when both dates were available and could be converted into valid calendar dates.

Reports were excluded from the TTO analysis if the treatment start date or event onset date was missing, partially recorded, internally inconsistent, or implausible. Negative TTO values, indicating an event date earlier than the recorded treatment start date, were excluded. Extremely long intervals were reviewed and excluded when they were considered clinically implausible or likely to reflect data-entry errors. Reports with valid TTO values were analyzed separately for overall adverse events, gastrointestinal adverse events, and metabolism/nutrition-related adverse events.

Because TTO information in spontaneous reporting systems is often incomplete, the Weibull distribution analysis was considered exploratory. The number of reports with valid TTO data was reported for each analysis group, and the proportion was reported where the corresponding denominator was available. The early reporting pattern was interpreted cautiously and was not considered evidence of a precise incidence curve or causal time-risk relationship. Sensitivity analyses based on alternative date-quality restrictions should be considered in future studies using more complete longitudinal data.

### 2.5 Toxicological analysis

To explore potential mechanistic clues related to metabolism- and nutrition-related disorders reported for azacitidine and decitabine, an exploratory network toxicology analysis was performed. Drug-related targets of azacitidine and decitabine were retrieved from PubChem and SwissTargetPrediction and then integrated after removal of duplicate entries. Genes potentially associated with metabolism- and nutrition-related disorders were collected from GeneCards and the Comparative Toxicogenomics Database (CTD). The overlapping genes between drug-related targets and disorder-related genes were identified as candidate common targets and visualized using a Venn diagram.

The candidate common targets were subsequently imported into the STRING database to construct a protein-protein interaction (PPI) network. Cytoscape 3.10.4 was used for network visualization and further screening of candidate hub targets. To explore the biological relevance of these candidate targets, Gene Ontology (GO) and Kyoto Encyclopedia of Genes and Genomes (KEGG) pathway enrichment analyses were conducted using the DAVID database. GO enrichment analysis included biological process (BP), cellular component (CC), and molecular function (MF) categories, whereas KEGG enrichment analysis was used to identify potentially involved biological pathways. Enriched terms with a multiple-testing-corrected *P* value < 0.05 and a gene count ≥3 were considered statistically significant. Given the exploratory nature of this analysis, the network toxicology results were interpreted as hypothesis-generating evidence rather than direct mechanistic validation. The overall workflow of data extraction, processing, and analysis is shown in Figure 1.

## 3 Results

### 3.1 Baseline characteristics of the study population

As shown in Table 1, a total of 14,216 azacitidine-related and 3,591 decitabine-related adverse event reports were included in the FAERS database. Azacitidine accounted for a larger number of reports than decitabine. For both drugs, reports were more frequently submitted for male patients than for female patients. Among azacitidine-related reports, 7,556 cases (53.2%) involved male patients and 4,735 cases (33.3%) involved female patients, with sex information missing in 1,925 reports (13.5%). Among decitabine-related reports, 1,758 cases (49.0%) involved male patients and 1,305 cases (36.3%) involved female patients, with missing sex information in 528 reports (14.7%).

**Table 1:** Baseline characteristics of adverse event reports associated with the hypomethylating agents azacitidine and decitabine.

Characteristics	Azacitidine		Decitabine	
Number of events	14,216		3,591	
Sex, *n* (%)	–		–	
Female	4,735 (33.3%)		1,305 (36.3%)	
Male	7,556 (53.2%)		1,758 (49.0%)	
Missing	1,925 (13.5%)		528 (14.7%)	
Age, number (%)	–		–	
< 18	277 (1.9%)		124 (3.5%)	
18–64.9	2,689 (18.9%)		782 (21.8%)	
65–85	7,641 (53.7%)		1,478 (41.2%)	
>85	422 (3.0%)		99 (2.8%)	
Missing	3,187 (22.4%)		1,108 (30.9%)	
Body weight, kg, *n* (%)	–		–	
< 50	407 (2.9%)		51 (1.4%)	
50–100	4,107 (28.9%)		810 (22.6%)	
≥100	309 (2.2%)		90 (2.5%)	
Missing	9,393 (66.1%)		2,640 (73.5%)	
**Top 5 reported countries, number (%)**	United States	4,081 (28.71%)	United States	2,070 (57.64%)
	Japan	1,637 (11.52%)	Canada	434 (12.09%)
	France	972 (6.84%)	China	364 (10.14%)
	Canada	891 (6.27%)	Italy	109 (3.04%)
	Spain	748 (5.26%)	Germany	85 (2.37%)

The age distribution was mainly concentrated in adult and older populations (Table 1). For azacitidine, the largest age group was 65–85 years, accounting for 7,641 reports (53.7%), followed by patients aged 18–64.9 years (2,689 reports, 18.9%), those aged >85 years (422 reports, 3.0%), and those aged < 18 years (277 reports, 1.9%). Age information was missing in 3,187 azacitidine-related reports (22.4%). For decitabine, reports were also most frequently observed in patients aged 65–85 years (1,478 reports, 41.2%), followed by those aged 18–64.9 years (782 reports, 21.8%), < 18 years (124 reports, 3.5%), and >85 years (99 reports, 2.8%). Age information was missing in 1,108 decitabine-related reports (30.9%).

Body weight data were frequently missing for both drugs, particularly for decitabine (Table 1). Among azacitidine-related reports, 4,107 cases (28.9%) involved patients weighing 50–100 kg, 407 cases (2.9%) involved patients weighing < 50 kg, and 309 cases (2.2%) involved patients weighing ≥100 kg, while body weight was missing in 9,393 reports (66.1%). Among decitabine-related reports, 810 cases (22.6%) involved patients weighing 50–100 kg, 51 cases (1.4%) involved patients weighing < 50 kg, and 90 cases (2.5%) involved patients weighing ≥100 kg, with missing body weight information in 2,640 reports (73.5%). Therefore, body weight-related findings should be interpreted with caution.

**Figure 2:**
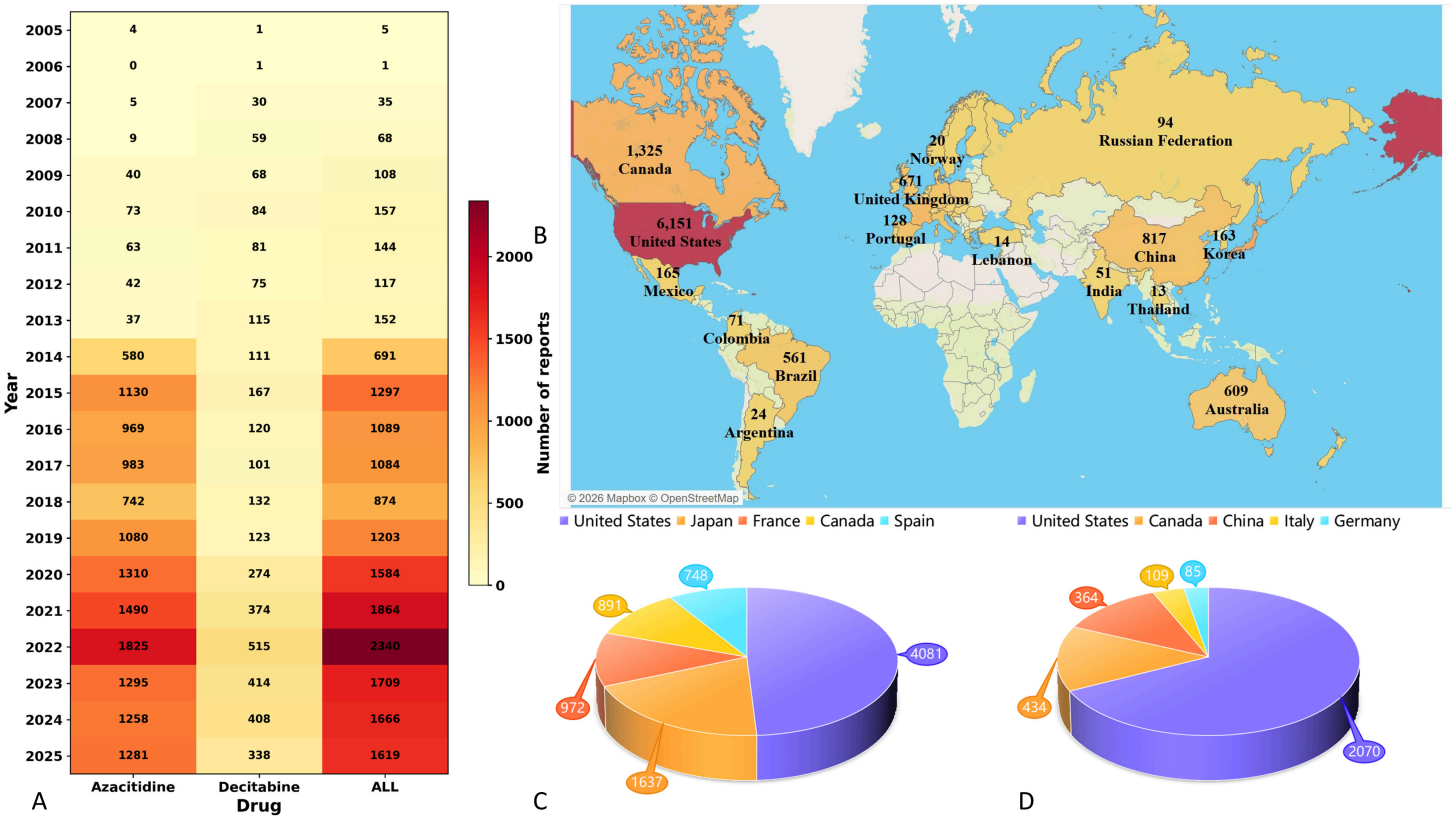
Temporal and spatial distribution of adverse events for hypomethylating agents azacitidine and decitabine. **(A)** Distribution of adverse event reporting times; **(B)** distribution of adverse event reporting by country/region; **(C)** top five countries reporting azacitidine; **(D)** top five countries reporting decitabine.

Regarding geographic distribution, the United States was the leading reporting country for both drugs (Figures 2B–D and Table 1), contributing 4,081 azacitidine-related reports (28.71%) and 2,070 decitabine-related reports (57.64%). Other major reporting countries were Japan, France, Canada, and Spain for azacitidine (Figure 2C), and Canada, China, Italy, and Germany for decitabine (Figure 2D).

As illustrated in Figure 2A, the annual number of adverse event reports for both drugs generally increased from 2005 to 2025, with relatively low reporting before 2014 and a marked increase thereafter. Azacitidine-related reports exceeded 1,000 cases in 2015 and reached a peak of 1,825 cases in 2022. Decitabine-related reports were consistently fewer than azacitidine-related reports but showed a similar upward trend, reaching a peak of 515 cases in 2022. The total number of reports also peaked in 2022, with 2,340 reports, followed by a slight decline from 2023 to 2025 while remaining at a relatively high level. Overall, azacitidine accounted for the majority of reports and was the main contributor to the temporal increase in reporting.

### 3.2 SOC signal analysis

**Figure 3:**
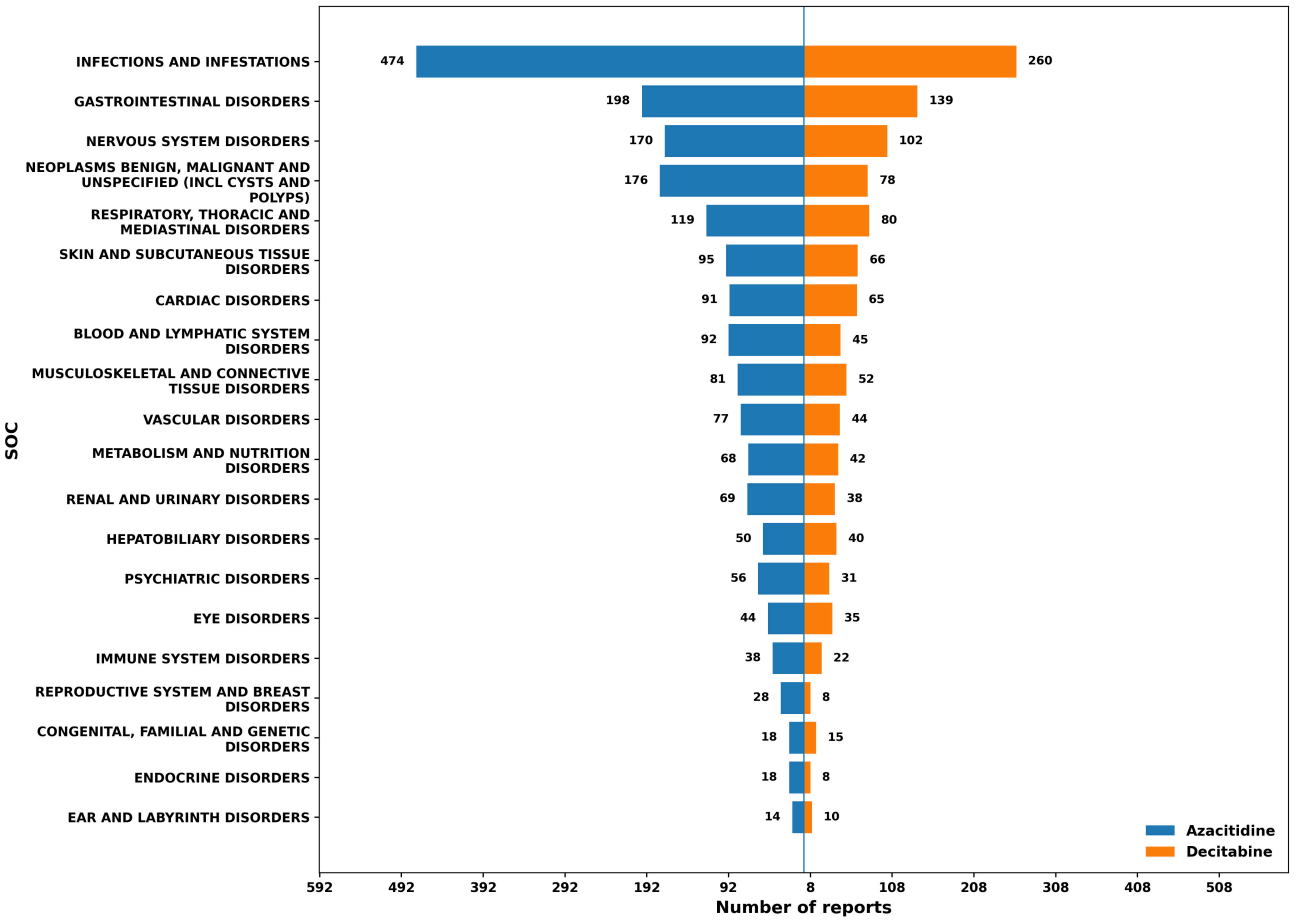
SOC-level adverse event signals associated with the hypomethylating agents azacitidine and decitabine.

As shown in Figure 3, the SOC-level distribution of positive adverse event signals was broadly similar between azacitidine and decitabine. In this analysis, the values represent the number of positive preferred-term signals mapped to each primary SOC, rather than the total number of adverse event reports within that SOC. For both drugs, Infections and infestations contained the largest number of mapped positive signals, with 474 signal entries for azacitidine and 260 for decitabine, yielding a total of 734 signal entries. Gastrointestinal disorders also ranked prominently, with 198 signal entries for azacitidine and 139 for decitabine, for a total of 337 signal entries, making it one of the leading non-infectious SOC categories. Nervous system disorders, Neoplasms benign, malignant and unspecified (including cysts and polyps), and Respiratory, thoracic and mediastinal disorders also showed relatively high numbers of mapped signals, suggesting that adverse event reporting signals associated with these two agents involved multiple organ systems.

Metabolism and nutrition disorders accounted for 68 signal entries for azacitidine and 42 for decitabine, with a total of 110 signal entries. Although this SOC was not among the highest-ranking categories, it was comparable to Renal and urinary disorders, which included 107 signal entries. As illustrated by the butterfly plot, azacitidine generally showed a larger number of mapped signals than decitabine across most SOCs, while the ranking of major affected systems was broadly consistent between the two drugs. These findings suggest that azacitidine and decitabine shared a generally similar SOC-level signal distribution, although differences in the number of mapped signals were observed. Given the clinical relevance of gastrointestinal symptoms and metabolism- and nutrition-related abnormalities in patients receiving hypomethylating agents, these two SOC categories were selected for further preferred-term-level characterization.

### 3.3 Signal analysis of gastrointestinal adverse reactions

**Figure 4:**
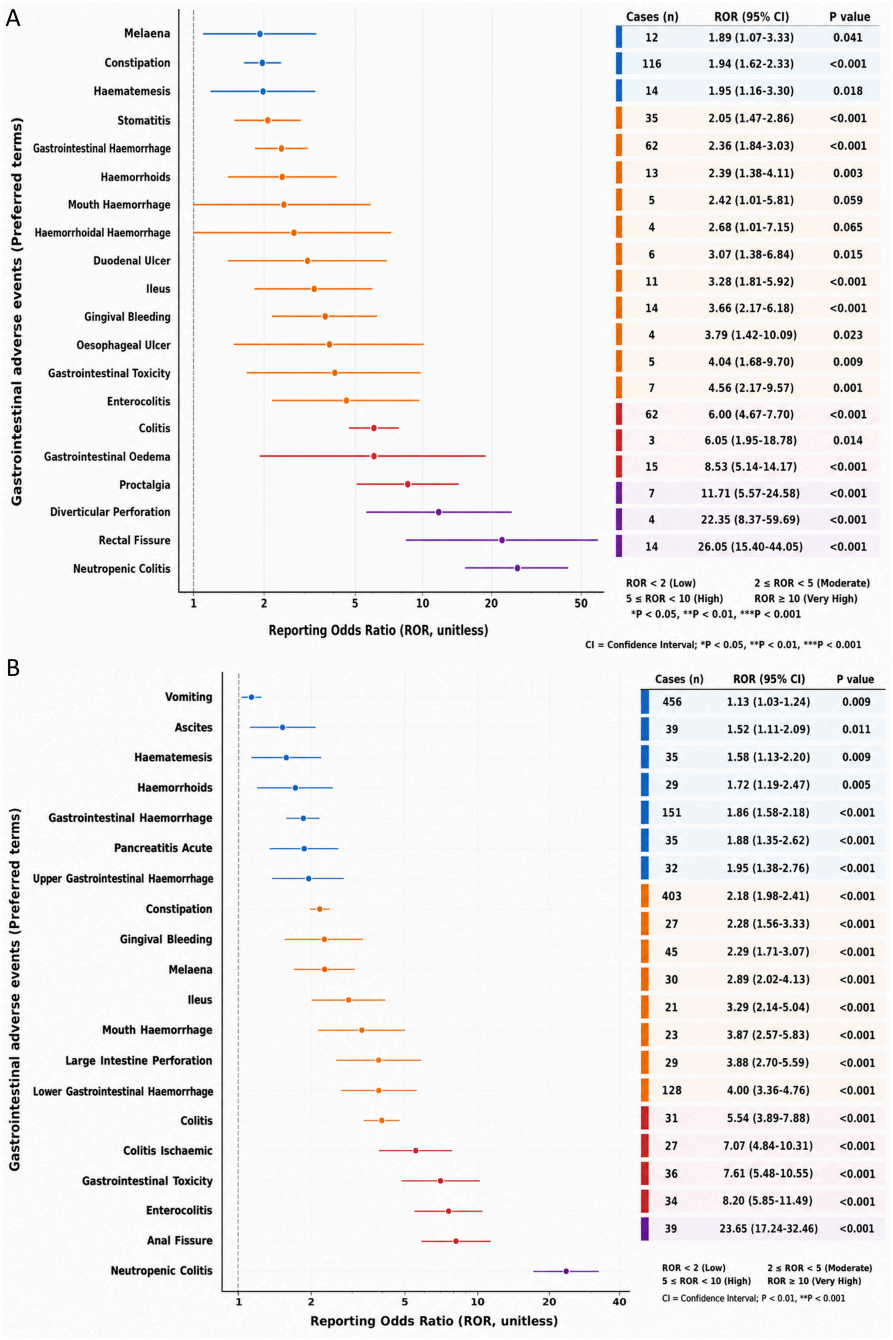
Forest plots of hypomethylating agent-related gastrointestinal adverse reaction signals based on the FAERS database. **(A)** Top 20 gastrointestinal PT signals associated with azacitidine. **(B)** Top 20 gastrointestinal PT signals associated with decitabine. Dots represent the reporting odds ratio (ROR), and horizontal lines indicate the 95% confidence interval (CI). Asterisks indicate statistical significance: ^*^p < 0.05, ^**^p < 0.01, and ^***^p < 0.001.

Figure 4A presents the top 20 gastrointestinal preferred-term (PT) signals associated with azacitidine. Among these PTs, neutropenic colitis showed the strongest disproportionality signal (ROR = 23.65, 95% CI: 17.24–32.46). Other PTs with relatively high ROR-based signal intensity included anal fissure (ROR = 8.20), enterocolitis (ROR = 7.61), gastrointestinal toxicity (ROR = 7.07), ischemic colitis (ROR = 5.54), and colitis (ROR = 4.00, *n* = 128). In contrast, vomiting (*n* = 456) and constipation (*n* = 403) had the largest numbers of reports but showed lower disproportionality estimates, with RORs of 1.13 and 2.18, respectively. These findings indicate that the gastrointestinal reporting profile of azacitidine included both frequently reported symptoms and less frequent but stronger disproportionality signals. Therefore, interpretation should consider both reporting frequency and signal intensity rather than relying on either measure alone.

Figure 4B presents the top 20 gastrointestinal PT signals associated with decitabine. Similar to azacitidine, neutropenic colitis showed the strongest disproportionality signal (ROR = 26.05, 95% CI: 15.40–44.05). Rectal fissure (ROR = 22.35) and diverticular perforation (ROR = 11.71) also showed relatively strong disproportionality signals. Additional PTs with notable signal intensity included proctalgia (ROR = 8.53), gastrointestinal edema (ROR = 6.05), and colitis (ROR = 6.00, *n* = 62). Constipation (*n* = 116) and gastrointestinal hemorrhage (*n* = 62) were reported relatively frequently but showed lower ROR values of 1.94 and 2.36, respectively. Mouth hemorrhage (*P* = 0.059) and hemorrhoidal hemorrhage (*P* = 0.065), although included among the plotted PTs, should be interpreted cautiously because their statistical evidence was borderline. Overall, the gastrointestinal reporting profile of decitabine was marked by strong disproportionality signals for neutropenic colitis, rectal fissure, and diverticular perforation, together with relatively frequent reports of constipation and gastrointestinal hemorrhage. These findings support the clinical relevance of monitoring gastrointestinal symptoms, particularly those suggestive of mucosal injury, inflammatory complications, or bleeding events in patients receiving decitabine.

### 3.4 Signal analysis of metabolism and nutrition disorders

**Figure 5:**
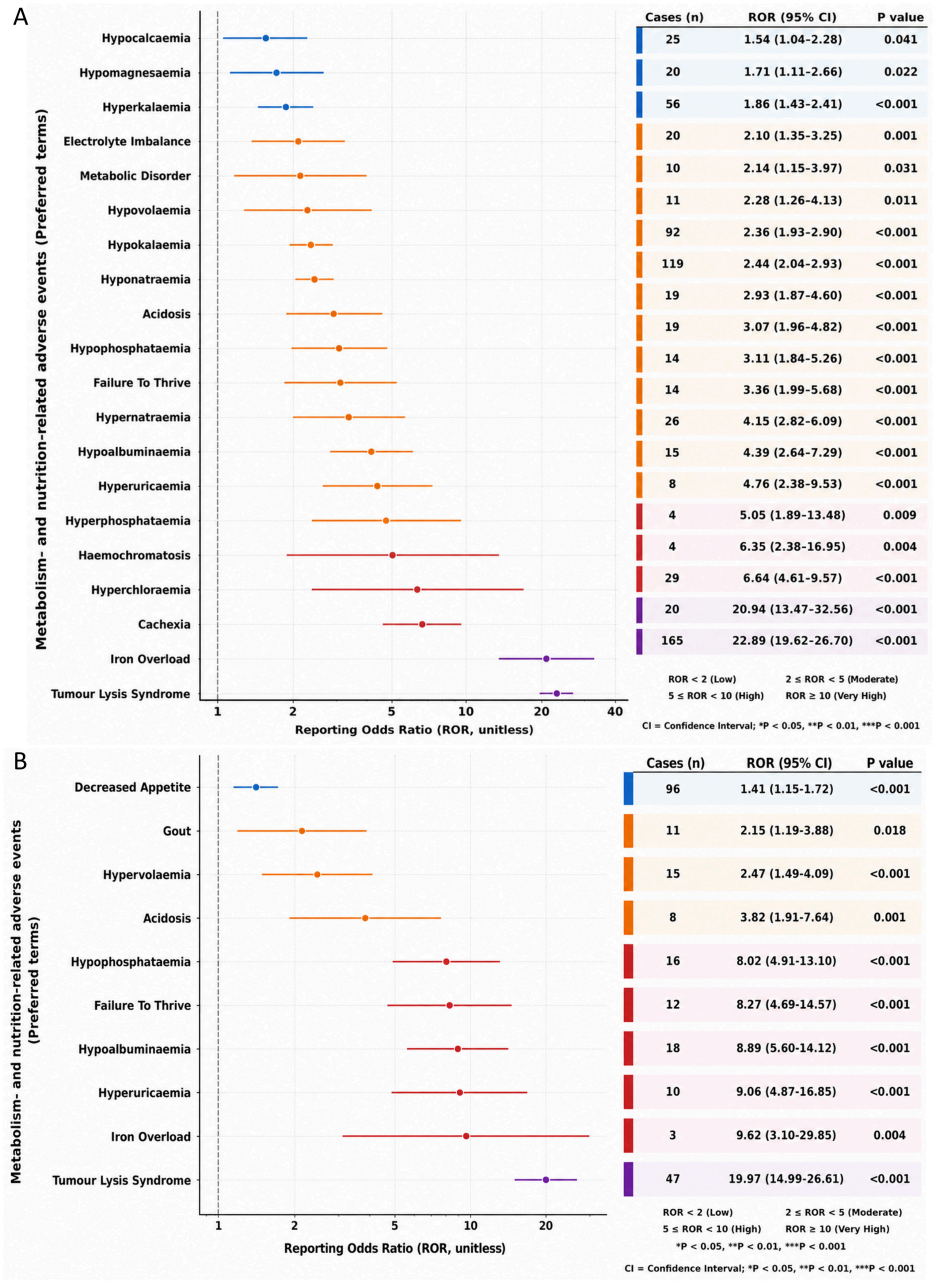
Forest plots of metabolism and nutrition disorder signals associated with hypomethylating agents based on the FAERS database. **(A)** Top 20 metabolism and nutrition disorder PT signals associated with azacitidine; **(B)** top 20 metabolism and nutrition disorder PT signals associated with decitabine. Dots indicate the reporting odds ratio (ROR), and horizontal lines indicate the 95% confidence interval (CI). Asterisks indicate statistical significance: ^*^p < 0.05, ^**^p < 0.01, and ^***^p < 0.001.

As shown in Figure 5A, among the metabolism- and nutrition-related preferred-term (PT) signals associated with azacitidine, tumor lysis syndrome showed the strongest disproportionality signal (ROR = 22.89, 95% CI: 19.62–26.70) and the largest number of reports (*n* = 165). Iron overload also showed a strong disproportionality signal (ROR = 20.94, 95% CI: 13.47–32.56). Additional PTs with relatively high ROR-based signal intensity included cachexia (ROR = 6.64), hyperchloremia (ROR = 6.35), and hemochromatosis (ROR = 5.05).

Several electrolyte and nutritional abnormalities were also identified. Hyponatremia (*n* = 119) and hypokalemia (*n* = 92) were among the most frequently reported PTs in this category, although their disproportionality estimates were lower, with ROR values of 2.44 and 2.36, respectively. Other metabolism- and nutrition-related PTs, including hypoalbuminemia (ROR = 4.15), hyperuricemia (ROR = 4.39), and hyperphosphatemia (ROR = 4.76), also showed positive reporting signals. Overall, the metabolism and nutrition reporting profile of azacitidine was characterized by strong disproportionality signals for tumor lysis syndrome and iron overload, together with more frequently reported electrolyte and nutritional abnormalities. These findings suggest that monitoring of tumor lysis indicators, electrolyte balance, albumin levels, and iron metabolism may be clinically relevant in patients receiving azacitidine, particularly during the early phase of treatment or in patients with high tumor burden, transfusion dependence, or poor baseline nutritional status.

As shown in Figure 5B, a total of 10 metabolism- and nutrition-related PT signals were identified for decitabine. Tumor lysis syndrome showed the strongest disproportionality signal (ROR = 19.97, 95% CI: 14.99–26.61) and a relatively high number of reports (*n* = 47). Other PTs with notable ROR-based signal intensity included iron overload (ROR = 9.62), hyperuricemia (ROR = 9.06), hypoalbuminemia (ROR = 8.89), failure to thrive (ROR = 8.27), and hypophosphatemia (ROR = 8.02). Among these PTs, hypoalbuminemia had 18 reports.

Additional metabolism- and nutrition-related signals included acidosis (ROR = 3.82), hypervolemia (ROR = 2.47), and gout (ROR = 2.15). Decreased appetite had the largest number of reports in this category (*n* = 96), but its disproportionality estimate was relatively low (ROR = 1.41), indicating that high reporting frequency does not necessarily correspond to strong disproportionality. Overall, the metabolism and nutrition reporting profile of decitabine was marked by a prominent disproportionality signal for tumor lysis syndrome, together with signals related to iron overload, hypoalbuminemia, hyperuricemia, failure to thrive, and phosphate abnormalities. These findings support the clinical relevance of monitoring tumor lysis-related laboratory indicators, nutritional status, electrolyte balance, and iron overload, especially in patients with advanced hematologic malignancies, transfusion dependence, or concomitant therapies that may increase metabolic vulnerability.

### 3.5 Time-to-onset analysis

**Figure 6:**
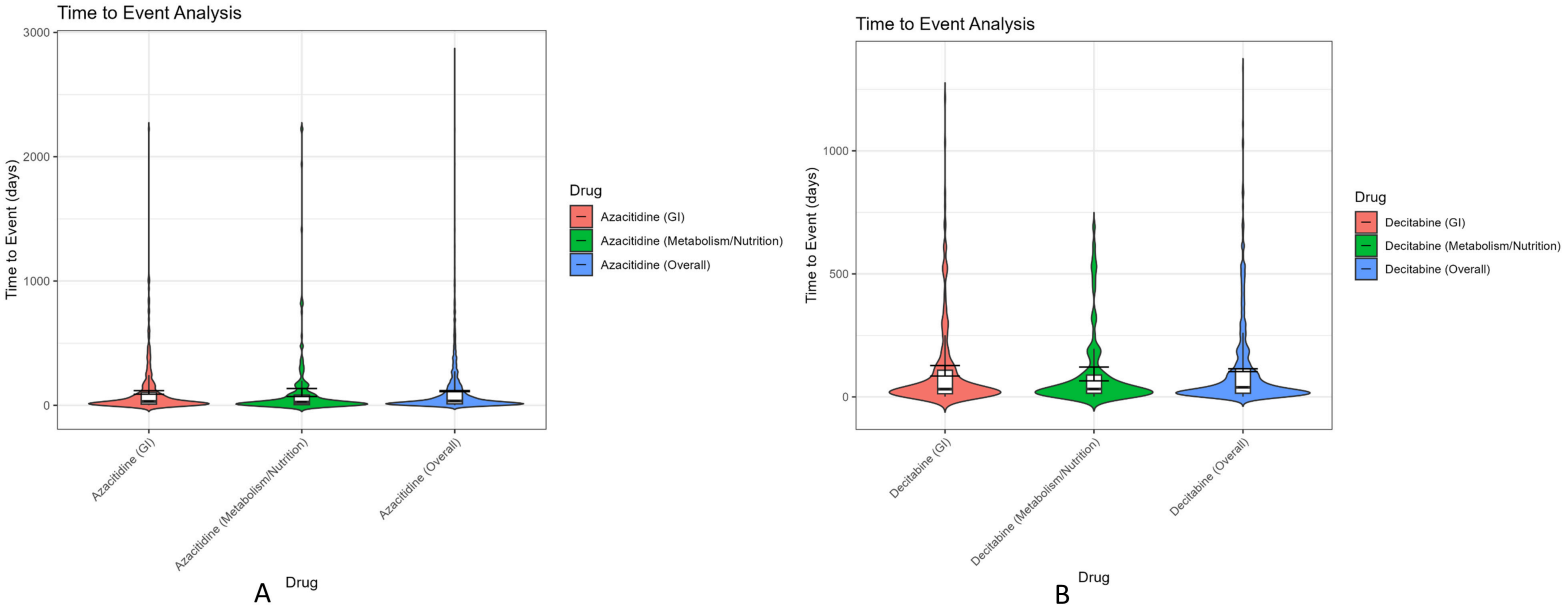
Time-to-onset analysis of adverse events associated with azacitidine and decitabine. **(A)** Violin plots of time to onset for azacitidine-related overall adverse events, gastrointestinal adverse events, and metabolism/nutrition-related adverse events; **(B)** violin plots of time to onset for decitabine-related overall adverse events, gastrointestinal adverse events, and metabolism/nutrition-related adverse events.

Time-to-onset (TTO) analysis was performed only for reports with valid treatment start dates and adverse event onset dates. Reports with missing, incomplete, implausible, or negative TTO values were excluded from this analysis. As shown in Figure 6 and Table 2, valid TTO information was available for 5,376 of 14,216 azacitidine-related overall adverse event reports (37.8%) and 3,419 of 3,591 decitabine-related overall adverse event reports (95.2%). For gastrointestinal adverse events, 791 azacitidine-related reports and 268 decitabine-related reports had valid TTO information. For metabolism/nutrition-related adverse events, valid TTO information was available for 270 azacitidine-related reports and 108 decitabine-related reports. Given the incomplete nature of date information in spontaneous reporting systems, the following results should be interpreted as exploratory temporal reporting patterns rather than precise estimates of event timing or incidence.

**Table 2:** Time to onset of azacitidine- and decitabine-related adverse events and Weibull distribution analysis.

Drug	TTO (days)		Weibull distribution		
	Case reports	Median (day)	Scale parameter: α (95%CI)	Shape parameter: β (95%CI)	Type
Azacitidine (overall)	5,376	47	95.05 (91.11–98.99)	0.68 (0.66–0.70)	Early reporting pattern
Azacitidine (GI)	791	31	70.57 (62.40–78.75)	0.62 (0.60–0.67)	Early reporting pattern
Azacitidine (metabolism/nutrition)	270	25	55.97 (43.54–68.41)	0.56 (0.52–0.61)	Early reporting pattern
Decitabine (overall)	3,419	39	84.89 (80.66–89.11)	0.71 (0.69–0.73)	Early reporting pattern
Decitabine (GI)	268	31.5	78.59 (64.02–93.16)	0.68 (0.62–0.74)	Early reporting pattern
Decitabine (metabolism/nutrition)	108	32	70.62 (50.25–90.99)	0.69 (0.59–0.79)	Early reporting pattern

TTO, time to onset; CI, confidence interval.

Among reports with valid TTO data, adverse events associated with both drugs tended to be reported relatively early after treatment initiation. For overall adverse events, the median TTO was 47 days for azacitidine and 39 days for decitabine. The Weibull scale parameter α was 95.05 (95% CI: 91.11–98.99) for azacitidine and 84.89 (95% CI: 80.66–89.11) for decitabine. The corresponding shape parameter β was 0.68 (95% CI: 0.66–0.70) and 0.71 (95% CI: 0.69–0.73), respectively, suggesting an early reporting pattern among cases with complete date information.

By event category, gastrointestinal adverse events also showed an early reporting tendency. The median TTO was 31 days for azacitidine-related gastrointestinal adverse events and 31.5 days for decitabine-related gastrointestinal adverse events. The α values were 70.57 (95% CI: 62.40–78.75) and 78.59 (95% CI: 64.02–93.16), respectively, and the β values were 0.62 (95% CI: 0.60–0.67) and 0.68 (95% CI: 0.62–0.74), respectively. These findings suggest that gastrointestinal adverse events were more frequently reported during the earlier phase of treatment among reports suitable for TTO analysis.

Metabolism/nutrition-related adverse events showed a similar exploratory temporal pattern. For azacitidine, the median TTO was 25 days, with an α value of 55.97 (95% CI: 43.54–68.41) and a β value of 0.56 (95% CI: 0.52–0.61). For decitabine, the median TTO was 32 days, with an α value of 70.62 (95% CI: 50.25–90.99) and a β value of 0.69 (95% CI: 0.59–0.79). Although these results suggest an early reporting tendency for gastrointestinal and metabolism/nutrition-related events, they should not be interpreted as definitive evidence that these events cluster within the first treatment cycle. Rather, they indicate that closer monitoring during the early phase of HMA therapy may be clinically prudent, particularly for patients with high tumor burden, baseline cytopenias, infection risk, or concomitant venetoclax-based therapy.

### 3.6 Cross-database signal consistency using Canada Vigilance data

To examine whether gastrointestinal and metabolism/nutrition-related disproportionality signals observed in FAERS showed similar reporting patterns in another spontaneous reporting system, we performed a cross-database signal consistency analysis using the Canada Vigilance Adverse Reaction Online Database (CVARDD). A disproportionality analysis framework consistent with that used for FAERS was applied, incorporating four methods: ROR, PRR, MGPS, and BCPNN. A signal was considered detected if at least one method met the predefined threshold. Cross-database consistency was primarily defined as a positive ROR signal in both databases, with the lower limit of the 95% confidence interval greater than 1 and at least three reports in each database. Because both FAERS and CVARDD are spontaneous reporting systems, consistent signals across the two databases were interpreted as reproducible reporting patterns rather than as evidence of incidence, absolute risk, or causal association.

For azacitidine, 21 positive signals within the target SOCs were identified in FAERS, of which 12 showed cross-database consistency in CVARDD. Among gastrointestinal disorders, colitis (FAERS ROR = 4.00; CVARDD ROR = 8.71), neutropenic colitis (FAERS ROR = 23.65; CVARDD ROR = 187.93), and ileus (FAERS ROR = 2.89; CVARDD ROR = 25.48) showed consistent disproportional reporting across the two databases. Among metabolism and nutrition disorders, tumor lysis syndrome (FAERS ROR = 22.89; CVARDD ROR = 33.78) and hypokalaemia (FAERS ROR = 2.36; CVARDD ROR = 8.23) also showed similar reporting patterns.

For decitabine, 58 positive signals within the target SOCs were detected in FAERS, of which eight showed cross-database consistency in CVARDD. Constipation (FAERS ROR = 1.94; CVARDD ROR = 4.31), stomatitis (FAERS ROR = 2.05; CVARDD ROR = 6.14), and gingival bleeding (FAERS ROR = 3.66; CVARDD ROR = 9.26) showed consistent disproportional reporting in both databases. Among metabolism and nutrition disorders, tumor lysis syndrome (FAERS ROR = 19.97; CVARDD ROR = 14.14) and iron overload (FAERS ROR = 9.62; CVARDD ROR = 46.86) showed similar cross-database reporting patterns. Notably, tumor lysis syndrome showed consistent disproportional reporting for both azacitidine and decitabine, suggesting a potential class-related reporting pattern that warrants clinical attention during HMA therapy.

In summary, several gastrointestinal and metabolism/nutrition-related signals for azacitidine and decitabine showed consistent disproportional reporting in FAERS and CVARDD. These findings support the reproducibility of selected reporting patterns across two spontaneous reporting systems, but they should be interpreted as hypothesis-generating pharmacovigilance evidence rather than confirmation of causality or absolute risk. The complete cross-database consistency results are presented in Supplementary Table S2.

### 3.7 Toxicological analysis

**Figure 7:**
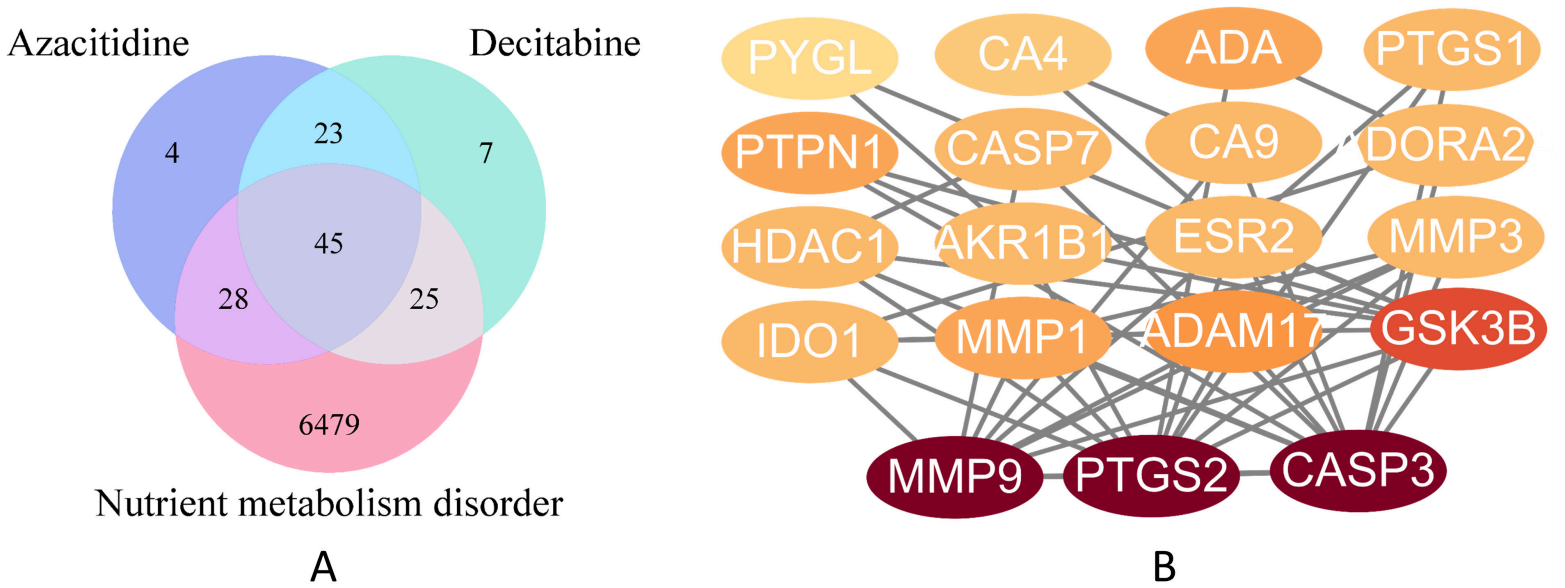
Potential common targets and protein interaction network underlying metabolism and nutrition disorders associated with azacitidine and decitabine. **(A)** Venn diagram of common targets among azacitidine, decitabine, and metabolism and nutrition disorders. **(B)** PPI network analysis of the common targets.

As shown in Figure 7A, a total of 45 overlapping targets were identified among azacitidine, decitabine, and metabolism and nutrition disorders. In addition to the targets shared by all three, 23 targets were common to azacitidine and decitabine but did not overlap with the disease targets. The numbers of specific overlapping targets between azacitidine and the disease and between decitabine and the disease were 28 and 25, respectively. Moreover, azacitidine and decitabine had 4 and 7 unique targets, respectively, whereas metabolism and nutrition disorders had 6,479 unique targets. These findings indicate that the toxicological mechanisms underlying metabolism and nutrition disorders associated with the two hypomethylating agents are supported by both shared biological foundations and distinct molecular characteristics. A protein-protein interaction (PPI) network was further constructed using the 45 intersecting genes. As shown in Figure 7B, extensive protein interactions were observed among these targets. According to the network topology, MMP9, PTGS2, CASP3, GSK3B, and ADAM17 occupied central positions in the network, among which MMP9, PTGS2, and CASP3 showed darker node colors and denser connections, suggesting that they may serve as core hub genes. These results suggest that azacitidine- and decitabine-related metabolism and nutrition disorders may involve the coordinated participation of multiple biological processes, including inflammatory regulation, apoptotic signaling, and matrix remodeling.

**Figure 8:**
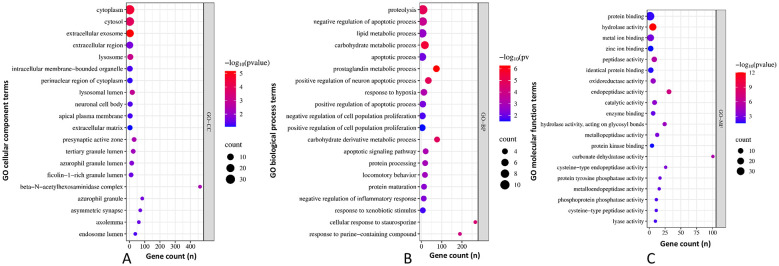
GO enrichment analysis of common targets associated with metabolism and nutrition disorders related to azacitidine and decitabine. **(A)** Biological process (GO-BP) enrichment analysis. **(B)** Cellular component (GO-CC) enrichment analysis. **(C)** Molecular function (GO-MF) enrichment analysis.

The GO biological process (GO-BP) enrichment analysis, shown in Figure 8A, indicated that the intersecting targets were mainly involved in proteolysis, regulation of apoptosis, and metabolism-related biological processes. Significantly enriched terms included proteolysis, negative regulation of apoptotic process, carbohydrate metabolic process, lipid metabolic process, and prostaglandin metabolic process, suggesting that these targets may contribute to the development of related toxic effects primarily by affecting protein degradation, apoptosis, and abnormalities in glucose and lipid metabolism. At the same time, processes such as response to hypoxia, negative regulation of inflammatory response, and response to xenobiotic stimulus also showed a certain degree of enrichment, indicating that inflammatory regulation, hypoxic response, and responses to exogenous stimuli may likewise play important roles in the underlying mechanisms. Overall, the GO-BP analysis suggests that metabolism and nutrition abnormalities associated with azacitidine and decitabine may involve the combined participation of multiple biological processes, including metabolic disturbance, dysregulated apoptosis, and abnormal stress responses.

The GO cellular component (GO-CC) enrichment analysis, shown in Figure 8B, demonstrated that the intersecting targets were mainly localized to the cytoplasm and related subcellular structures. Among these, cytoplasm, cytosol, and extracellular exosome showed the most prominent enrichment, indicating that these targets are mainly distributed in intracellular fluid environments and extracellular vesicle-related structures. In addition, terms such as extracellular region, lysosome, and lysosomal lumen also exhibited relatively high enrichment, suggesting that lysosome-related structures and the extracellular microenvironment may be important in the relevant biological processes. Some targets were also enriched in cellular components such as presynaptic active zone, neuronal cell body, asymmetric synapse, and axolemma, implying possible involvement of neuron-related cellular structures. Furthermore, extracellular matrix, endosome lumen, and several granule lumen-related terms also showed a certain degree of enrichment. Overall, the GO-CC analysis indicates that the potential targets involved in azacitidine- and decitabine-related metabolism and nutrition abnormalities are mainly concentrated in the cytoplasm, extracellular vesicles, and lysosome-related structures, and may also involve extracellular transport and intracellular degradation processes.

The GO molecular function (GO-MF) enrichment analysis, shown in Figure 8C, revealed that the molecular functions of the intersecting targets were mainly concentrated in protein binding, enzymatic activity, and proteolysis-related functions. Among these, protein binding involved the largest number of genes (39), suggesting extensive protein interactions among these targets. Hydrolase activity (24 genes) and metal ion binding (20 genes) also showed substantial enrichment, indicating that hydrolytic enzyme activity and metal ion-dependent functions may play important roles in this molecular network. Meanwhile, terms such as peptidase activity, oxidoreductase activity, and endopeptidase activity were also significantly enriched, suggesting that protein degradation and processing, redox regulation, and endopeptidase activity may participate in the relevant toxic processes. In terms of enrichment intensity, carbonate dehydratase activity, endopeptidase activity, and cysteine-type endopeptidase activity showed relatively high enrichment values, indicating that some specific enzymatic functions were more prominently enriched among the intersecting targets. Overall, the GO-MF analysis suggests that the potential molecular mechanisms underlying metabolism and nutrition disorders associated with these two drugs may be closely related to abnormalities in protein binding, catalytic hydrolysis, metal ion binding, and protease activity regulation.

**Figure 9:**
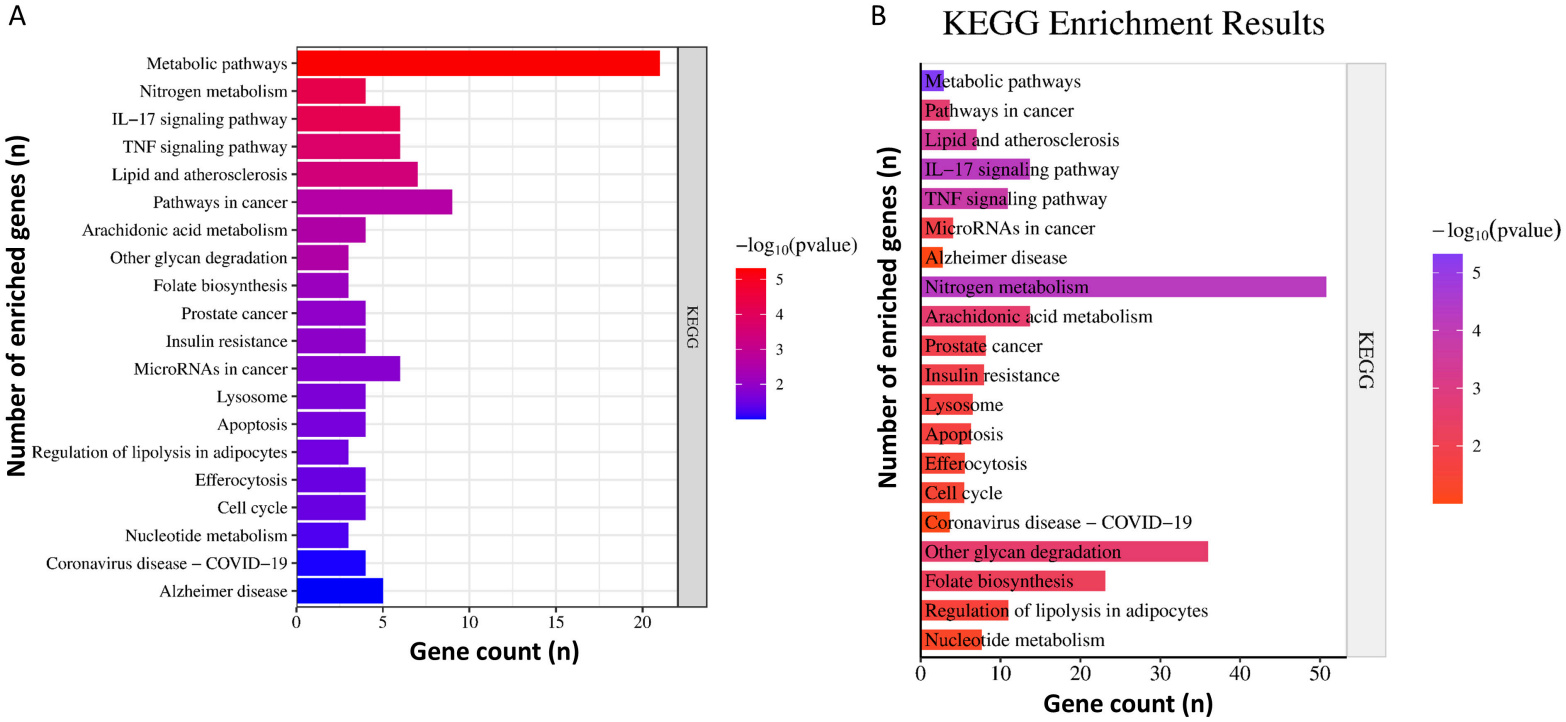
KEGG pathway enrichment analysis of common targets associated with metabolism and nutrition disorders related to azacitidine and decitabine. **(A)** KEGG pathway enrichment analysis based on gene count. **(B)** KEGG pathway enrichment analysis based on enrichment magnitude.

The KEGG pathway enrichment analysis shown in Figure 9A indicated that the intersecting targets were mainly enriched in Metabolic pathways, involving 21 targets, which was the pathway with the largest number of enriched genes. This finding suggests that metabolic abnormalities may constitute an important biological basis of metabolism and nutrition disorders associated with azacitidine and decitabine. In addition, pathways such as Lipid and atherosclerosis, IL-17 signaling pathway, and TNF signaling pathway also showed relatively high levels of enrichment, suggesting that disordered lipid metabolism and inflammatory responses may play key roles in the related toxic mechanisms. Meanwhile, several metabolism-related pathways, including Arachidonic acid metabolism, Nitrogen metabolism, Insulin resistance, and Regulation of lipolysis in adipocytes, were also enriched, further supporting a close association between these drug-related adverse reactions and disturbances in energy metabolism, lipid metabolism, and nutritional homeostasis.

Figure 9B further illustrates the KEGG pathway analysis results from the perspective of enrichment magnitude. The results showed that the intersecting targets exhibited the highest enrichment in Nitrogen metabolism (enrichment = 50.78), followed by other glycan degradation (35.97) and Folate biosynthesis (23.12), suggesting that these relatively specific metabolism-related pathways may have strong biological relevance to azacitidine- and decitabine-related metabolism and nutrition abnormalities. In addition, the IL-17 signaling pathway, Arachidonic acid metabolism, and TNF signaling pathway also showed relatively high enrichment levels, indicating that inflammatory regulation and abnormalities in lipid mediator metabolism may be involved in the toxic process. At the same time, pathways such as Insulin resistance, Regulation of lipolysis in adipocytes, Apoptosis, and Lysosome also reached a certain degree of enrichment, suggesting that dysregulation of glucose and lipid metabolism, apoptosis, and lysosome-related processes may jointly contribute to the occurrence of the relevant adverse reactions.

## 4 Discussion

### 4.1 Baseline characteristics of the study population

Based on real-world data from the FAERS database between 2005 and 2025, this study included 14,216 azacitidine-related reports and 3,591 decitabine-related reports. The larger number of azacitidine-related reports may reflect its broader clinical use, longer accumulated exposure, differences in reporting practices, and the expansion of HMA-based treatment strategies in patients with MDS and AML.

Regarding sex distribution, reports for both drugs were more frequently submitted for male patients. Among azacitidine-related reports, male patients accounted for 53.2% and female patients accounted for 33.3%, while sex information was missing in 13.5% of reports. Similarly, among decitabine-related reports, male patients accounted for 49.0% and female patients accounted for 36.3%, with missing sex information in 14.7% of reports. This male predominance is broadly consistent with the epidemiological characteristics of MDS and AML, which are more commonly diagnosed in older men (17, 18). Nevertheless, because FAERS is a spontaneous reporting system, these sex distributions should be interpreted as reporting patterns rather than true sex-specific incidence or exposure rates.

The age distribution also indicated that reports associated with both drugs were mainly concentrated in older adults. The median ages were 72 years for azacitidine and 70 years for decitabine, which is consistent with the typical age distribution of patients with MDS and AML (19). In the azacitidine group, patients aged 65–85 years accounted for the largest proportion of reports (53.7%), followed by those aged 18–64.9 years (18.9%), >85 years (3.0%), and < 18 years (1.9%). In the decitabine group, patients aged 65–85 years also represented the largest age category (41.2%), followed by those aged 18–64.9 years (21.8%), < 18 years (3.5%), and >85 years (2.8%). Age information was missing in 22.4% of azacitidine-related reports and 30.9% of decitabine-related reports. Therefore, although the overall pattern suggests that HMA-related reports were predominantly derived from older patients, age-related comparisons should be interpreted cautiously.

The reporting countries were predominantly the United States, accounting for 28.71% of azacitidine-related reports and 57.64% of decitabine-related reports. Azacitidine-related reports were also frequently submitted from Japan, France, Canada, and Spain, whereas decitabine-related reports were mainly submitted from Canada, China, Italy, and Germany. The relatively high proportion of reports from the United States is consistent with the intrinsic reporting structure of FAERS. The contribution of other countries may reflect regional differences in drug availability, clinical use, pharmacovigilance awareness, and reporting practices rather than true geographic differences in adverse event risk. In particular, the proportion of decitabine-related reports from China may be associated with its clinical accessibility and use in patients with MDS or AML (20).

Overall, the corrected baseline characteristics indicate that azacitidine generated more reports than decitabine, and that reports for both drugs were mainly concentrated in male and older patients. These characteristics provide an important descriptive context for interpreting subsequent signal analyses. However, because of spontaneous reporting bias and missing demographic information, the baseline distributions should not be interpreted as population-level exposure patterns or incidence estimates.

### 4.2 Gastrointestinal adverse reaction signals

Using four disproportionality analysis algorithms, this study systematically characterized the spectrum of gastrointestinal adverse reactions associated with the two drugs. The most notable finding was that neutropenic colitis showed the strongest signal for both azacitidine and decitabine, with ROR values of 23.65 and 26.05, respectively. A similar disproportional reporting pattern was observed in the Canada Vigilance Adverse Reaction Online Database (CVARDD), supporting cross-database signal consistency. Although this adverse reaction has been reported relatively infrequently in clinical trials, it showed an extremely high reporting odds ratio in real-world data.

The pathogenesis of neutropenic colitis, also known as ileocecal syndrome or typhlitis, involves the interaction of multiple factors (21). On the one hand, while hypomethylating agents exert antitumor effects by inhibiting DNA methyltransferases (DNMTs), they inevitably cause myelosuppression, leading to marked neutropenia and disruption of the intestinal mucosal immune barrier (22, 23). On the other hand, cytotoxic agents can directly damage the intestinal mucosal epithelium, resulting in mucosal edema, vasodilation, and mucosal surface disruption, thereby creating favorable conditions for bacterial invasion of the intestinal wall (24). Previous studies have reported that azacitidine use during AML induction chemotherapy is an independent risk factor for neutropenic colitis (OR = 2.45, 95% CI: 1.01–5.90) (25). The extremely high ROR values observed in the present study further confirm that this complication remains clinically relevant even under low-intensity hypomethylating treatment regimens and therefore warrants close clinical attention.

Among common gastrointestinal symptoms, vomiting and constipation had the highest numbers of reported cases but relatively low ROR values. This finding is generally consistent with the FDA-approved prescribing information, in which nausea, vomiting, diarrhea, and constipation are listed as the most common adverse reactions, with reported incidences exceeding 30%. Vascular events, such as colitis ischaemic (ROR = 5.54) and gastrointestinal hemorrhage, also deserve attention. Hypomethylating agents may affect vascular endothelial function and the coagulation system. In addition, older patients often have concomitant cardiovascular diseases, making them more susceptible to intestinal hypoperfusion under conditions of hypovolemia or hypotension.

### 4.3 Signals of metabolism and nutrition disorders

Although metabolism and nutrition disorders did not rank among the highest SOC categories in this study, signal intensity analysis revealed a series of metabolic toxicity signals with important clinical relevance. tumor lysis syndrome (TLS) showed the strongest enrichment signal for both drugs, with ROR values of 22.89 for azacitidine and 19.97 for decitabine, and was bidirectionally validated in CVARDD. This finding suggests that TLS represents the most prominent class-effect metabolic toxicity signal of hypomethylating agents, which is consistent with existing basic research evidence (26).

The occurrence of TLS may be related to the antitumor effects of hypomethylating agents and the baseline disease burden of patients (27). Both azacitidine and decitabine are nucleoside analog hypomethylating agents. Decitabine is primarily incorporated into DNA, whereas azacitidine is mainly incorporated into RNA and can be partially converted and incorporated into DNA. Both agents exert antitumor effects by forming covalent DNMT-DNA complexes, inhibiting DNA methylation, and regulating the proliferation, differentiation, and apoptosis of abnormal clonal cells (28). In patients with high tumor burden, active leukemic cell proliferation, or rapid treatment-induced cell death, particularly when combined with venetoclax, massive leukemic cell lysis within a short period may lead to the release of intracellular potassium, phosphorus, and nucleic acids. Nucleic acids are further catabolized into uric acid, ultimately causing characteristic metabolic abnormalities of TLS, including hyperuricemia, hyperkalemia, hyperphosphatemia, and secondary hypocalcemia (29). The FDA-approved prescribing information for azacitidine has been updated to include a warning for TLS, stating that the drug may cause fatal or serious TLS in patients with MDS. The signal detection results of the present study are highly consistent with this regulatory warning.

Iron overload was another prominent high-risk metabolic signal for both drugs, with ROR values of 20.94 for azacitidine and 9.62 for decitabine. Patients with MDS and AML are intrinsically at risk of iron overload because of ineffective hematopoiesis and long-term dependence on red blood cell transfusions (30). However, the significant aggregation of iron overload signals observed for both drugs in this study suggests that, beyond transfusion-related factors, hypomethylating agents may directly or indirectly affect systemic iron metabolism. Previous studies have shown that iron overload in MDS may aggravate bone marrow failure by impairing the function of bone marrow mesenchymal stromal cells and hematopoietic progenitor cells, while also increasing the risks of infection and cardiotoxicity (31). Gattermann reported that even before the development of transfusion dependence, ineffective hematopoiesis in patients with MDS may suppress hepatic hepcidin production, resulting in uncontrolled intestinal iron absorption. Hypomethylating agents may further interfere with the expression of iron metabolism-related genes through epigenetic regulation (32). Notably, both haemochromatosis (ROR = 5.05) and iron overload appeared in the azacitidine signal profile, suggesting that azacitidine-related disturbances in iron metabolism may involve hereditary haemochromatosis-like mechanisms or exacerbation of secondary iron deposition.

Cachexia in the azacitidine signal profile (ROR = 6.64) and failure to thrive in the decitabine signal profile (ROR = 8.27) reflect the potential negative effects of these drugs on systemic nutritional status. Cachexia is common in patients with cancer and involves an inflammatory cytokine-driven hypercatabolic state. Hypomethylating agents may aggravate this process by inducing cytokine release and DNA damage responses (33). In contrast, although decreased appetite had the highest number of reports for decitabine (*n* = 96), its ROR value was the lowest (1.41), suggesting that it may represent a relatively non-specific event.

### 4.4 Time-to-onset analysis

Time-to-onset analysis is an important dimension of pharmacovigilance research, as it can reveal temporal patterns of adverse event occurrence and guide the establishment of appropriate clinical monitoring windows. In this study, the Weibull distribution model was used to systematically characterize the temporal features of adverse events associated with azacitidine and decitabine. The results showed that the shape parameter β was less than 1 in all analyzed groups, indicating that adverse events associated with both drugs followed an early failure pattern, namely that the risk was highest during the initial phase of treatment and gradually decreased with prolonged exposure.

For overall adverse events, the median time to onset was 47 days for azacitidine and 39 days for decitabine, indicating that most events occurred within the first one to two treatment cycles, assuming a 28-day cycle. This pattern is consistent with clinical trial observations. The prescribing information for azacitidine explicitly states that adverse reactions such as thrombocytopenia, neutropenia, anemia, nausea, vomiting, and constipation are more pronounced during the first one to two treatment cycles (34). This early clustering phenomenon may be explained from a pharmacological perspective: the cytotoxic and epigenetic effects of hypomethylating agents are strongest during initial exposure. As treatment continues, sensitive clonal cells are eliminated, normal hematopoiesis and the mucosal epithelium gradually adapt, and the incidence of adverse events tends to decline.

By organ system, the median time to onset of gastrointestinal adverse events was approximately 31 days for both drugs, which was shorter than that of overall adverse events. The lower β value for azacitidine further suggests a stronger early-onset pattern of gastrointestinal toxicity. This phenomenon may be related to the rapid turnover cycle of intestinal mucosal epithelial cells, which is approximately 3–5 days. Direct drug-induced injury to intestinal stem cells and villous epithelial cells may rapidly impair mucosal barrier function, leading to diarrhea, colitis, bleeding, or other gastrointestinal symptoms within the first treatment cycle (35).

The early-onset pattern was even more pronounced for metabolism and nutrition disorder-related adverse events. For azacitidine, the median time to onset was only 25 days, and the β value was the lowest among all analyzed groups (0.56), indicating the strongest early failure tendency. This finding has important clinical warning value: TLS, electrolyte disturbances, and acute metabolic crises may occur as early as the first treatment cycle, requiring clinicians to complete risk assessment and implement preventive interventions before initiating hypomethylating therapy. In comparison, the β value for metabolism-related events associated with decitabine was 0.69, indicating a slightly less pronounced early-onset pattern than that observed for azacitidine, which may be related to differences in dosing regimens and pharmacokinetic characteristics.

### 4.5 Toxicological analysis

To explore the potential molecular mechanisms underlying hypomethylating agent-induced metabolism and nutrition disorders, this study established a network toxicology analysis framework. By integrating drug target prediction databases with disease-related gene databases, a total of 45 intersecting targets among azacitidine, decitabine, and metabolism and nutrition disorders were identified. PPI network analysis further identified several core hub genes, including MMP9, PTGS2 (COX-2), CASP3, GSK3B, and ADAM17.

MMP9 matrix metalloproteinase-9 and PTGS2 prostaglandin-endoperoxide synthase 2, also known as COX-2 occupied central positions in the network and showed the densest connections, suggesting that inflammatory regulation and matrix remodeling may represent key pathological processes in hypomethylating agent-related metabolic toxicity (36). MMP9 participates in extracellular matrix degradation, regulation of vascular permeability, and inflammatory cell infiltration. Its excessive activation can disrupt the integrity of the intestinal mucosal barrier and vascular basement membrane, and is closely associated with the occurrence of neutropenic colitis, gastrointestinal hemorrhage, and tissue oedema (37). PTGS2 is the rate-limiting enzyme in prostaglandin synthesis. It catalyzes the conversion of arachidonic acid to prostaglandin H2, which is further converted into mediators such as PGE2 with pro-inflammatory and vasodilatory effects. The significant enrichment of the arachidonic acid metabolism pathway in the KEGG analysis further supports this mechanism. Hypomethylating agents may indirectly upregulate the transcriptional expression of MMP9 and PTGS2 through DNA demethylation, or activate these inflammatory mediators by inducing cellular stress responses.

GO-BP enrichment analysis showed that the intersecting targets were significantly involved in biological processes such as proteolysis, negative regulation of apoptotic process, carbohydrate metabolic process, and lipid metabolic process. These findings, from the perspective of functional annotation, indicate that the metabolic toxicity of hypomethylating agents is not caused by disruption of a single pathway, but rather by dysregulation of networks involving protein degradation, energy metabolism, and cell death regulation (38). The enrichment of response to hypoxia also has important pathological implications. Anemia caused by myelosuppression, insufficient tissue perfusion, and inflammation-induced local hypoxia may activate the HIF-1α pathway, thereby further exacerbating metabolic stress.

GO-CC analysis indicated that the targets were mainly distributed in the cytoplasm, extracellular exosomes, and lysosome-related structures. As carriers of intercellular communication, extracellular vesicles can transport drug-induced injury signals and inflammatory mediators between organs, potentially contributing to distant effects and amplification of systemic metabolic disorders (39). The enrichment of lysosome-related components suggests that the autophagy-lysosome degradation system may play an important role in drug metabolism and the clearance of cellular damage.

KEGG pathway enrichment analysis further revealed the central role of Metabolic pathways, which involved 21 intersecting targets, suggesting that the metabolic toxicity of hypomethylating agents is characterized by broad perturbation of metabolic networks. The markedly high enrichment of Nitrogen metabolism enrichment = 50.78 suggests that the urea cycle and amino acid metabolism may be substantially affected, which is consistent with clinical manifestations such as hypoalbuminaemia and cachexia observed in this study (40). The enrichment of the IL-17 signaling pathway and TNF signaling pathway indicates that Th17 cell- and macrophage-mediated inflammatory responses may be important drivers of metabolic toxicity (41). Involvement of Insulin resistance and Regulation of lipolysis in adipocytes pathways further points to disruption of glucose and lipid metabolic homeostasis, which may partly explain the occurrence of TLS, electrolyte disturbances, and abnormalities in iron metabolism.

The enrichment of the Folate biosynthesis pathway provides a particularly important mechanistic clue. As nucleoside analogs, both azacitidine and decitabine are structurally based on a cytidine scaffold, whereas the folate cycle is a central process in one-carbon unit transfer and nucleotide synthesis. Hypomethylating agents may interfere with the regeneration of folate-dependent methyl donors, such as S-adenosylmethionine, thereby indirectly affecting one-carbon metabolism and nucleic acid synthesis and repair, resulting in a “metabolic antagonism” effect (42). This mechanism shares certain similarities with the toxicity mechanism of antifolate agents such as methotrexate, suggesting that folic acid or active folate derivatives may have potential protective effects, which warrants further experimental validation.

## 5 Limitations

This study has several limitations that should be considered when interpreting the findings. FAERS is a spontaneous reporting system and is therefore inherently affected by reporting bias, underreporting, duplicate reporting, and incomplete clinical information. Because the database does not provide the total number of patients exposed to azacitidine or decitabine, the true incidence or absolute risk of each adverse event could not be estimated. Accordingly, the disproportionality signals identified in this study should be interpreted as indicators of relative reporting frequency rather than as direct measures of causal association or incidence-based risk. Signals with high reporting odds ratios may reflect a potential drug-event association, but they may also be influenced by differential reporting behaviors, clinical awareness, regulatory attention, and the severity or recognizability of specific adverse events.

These biases may have affected the observed signal spectrum in different directions. Severe or clinically distinctive events, such as neutropenic colitis and tumor lysis syndrome, are more likely to be recognized and reported, which may strengthen their apparent disproportionality signals in FAERS. In contrast, mild, common, or expected symptoms, such as decreased appetite, nausea, vomiting, or constipation, may be underreported in routine practice, leading to an underestimation of their reporting frequency or signal strength. Reporting patterns may also vary over time and across countries, particularly with changes in prescribing practices, the introduction of new formulations, increasing use of venetoclax-based combination regimens, and updates to drug labels or safety warnings. Therefore, temporal and geographic differences in reporting should not be interpreted simply as differences in pharmacological risk.

Missing data represent another important limitation. In the present dataset, age and body weight information were unavailable for a considerable proportion of reports, especially body weight, which limited reliable age- or weight-stratified analyses. In addition, FAERS contains limited information on dose, treatment cycle, treatment duration, laboratory findings, tumor burden, disease stage, transfusion history, baseline nutritional status, and concomitant medications. These missing or incompletely reported variables restrict the ability to adjust for clinically important confounders. This issue is particularly relevant for HMAs, because patients receiving azacitidine or decitabine often have underlying MDS or AML, baseline cytopenias, infection susceptibility, nutritional vulnerability, transfusion dependence, and frequent exposure to combination therapies, including venetoclax. Many of these factors may independently contribute to gastrointestinal adverse events and metabolism- or nutrition-related disorders. Thus, the detected signals should not be attributed solely to azacitidine or decitabine.

Although similar disproportional reporting patterns in the Canada Vigilance Adverse Reaction Online Database supported the reproducibility of selected signals, this cross-database consistency should not be interpreted as causal validation and could not eliminate biases shared by spontaneous reporting systems. The results of this study should therefore be regarded as hypothesis-generating pharmacovigilance evidence. Further validation using prospective cohorts, electronic health record databases, claims data, or mechanistic experimental studies is needed to clarify the causal relationships, estimate absolute risks, and identify patient subgroups at higher risk for gastrointestinal and metabolic/nutritional toxicities during HMA therapy.

## 6 Conclusion

Based on FAERS data and cross-database signal consistency analysis using CVARDD, this study characterized disproportional reporting patterns of gastrointestinal adverse reactions and metabolism/nutrition disorders associated with the hypomethylating agents azacitidine and decitabine. Neutropenic colitis and tumor lysis syndrome emerged as prominent disproportionality signals for gastrointestinal and metabolism/nutrition-related events, respectively, and showed consistent reporting patterns across the two spontaneous reporting systems. Time-to-onset analysis suggested an early reporting pattern for adverse events associated with both drugs, with gastrointestinal and metabolism/nutrition-related events more frequently reported during the early treatment period. These findings support the clinical need for close monitoring of gastrointestinal symptoms, metabolic abnormalities, nutritional status, and tumor lysis-related indicators during initial treatment cycles. Network toxicology analysis provided exploratory mechanistic clues involving inflammatory regulation, apoptotic signaling, and metabolic pathway dysregulation. Overall, these findings should be interpreted as hypothesis-generating pharmacovigilance evidence rather than proof of incidence, absolute risk, or causality, and further validation using prospective clinical cohorts, electronic health record databases, and mechanistic studies is warranted.

## Data Availability

The original contributions presented in the study are included in the article/Supplementary material, further inquiries can be directed to the corresponding authors.
