# Supplementary material for: Gastrointestinal adverse reactions and metabolism–nutrition disorders associated with hypomethylating agents: a pharmacovigilance study with exploratory mechanistic analysis
Source: Front Nutr. 2026 Jun 30;13:1865637. doi: 10.3389/fnut.2026.1865637 (PMC13364762; doi:10.3389/fnut.2026.1865637)
Supplement: Supplementary file 2 [file Table_2.docx]

**Supplementary_Table2:** Full cross-database validation results of gastrointestinal and metabolic adverse events associated with azacitidine and decitabine between FAERS and CVARDD.

| **Drug** | **Preferred Term (PT)** | **SOC** | **FAERS Case (n)** | **FAERS ROR (95% CI)** | **FAERS Signal*** | **CVARDD Case (n)** | **CVARDD ROR (95% CI)** | **CVARDD Signal*** |
| --- | --- | --- | --- | --- | --- | --- | --- | --- |
| AZACITIDINE | Colitis | GI | 128 | 4.00 (3.36–4.76) | 4/4 | 4 | 8.71 (3.26–23.29) | 4/4 |
| AZACITIDINE | Ascites | GI | 39 | 1.52 (1.11–2.09) | 2/4 | 1 | 4.40 (0.62–31.31) | 1/4 |
| AZACITIDINE | Neutropenic Colitis | GI | 39 | 23.65 (17.24–32.46) | 4/4 | 2 | 187.93 (45.90–769.49) | 2/4 |
| AZACITIDINE | Haematemesis | GI | 35 | 1.58 (1.13–2.20) | 2/4 | 1 | 3.75 (0.53–26.66) | 1/4 |
| AZACITIDINE | Upper Gastrointestinal Haemorrhage | GI | 32 | 1.95 (1.38–2.76) | 2/4 | 1 | 5.69 (0.80–40.48) | 1/4 |
| AZACITIDINE | Ileus | GI | 30 | 2.89 (2.02–4.13) | 4/4 | 1 | 25.48 (3.57–181.90) | 2/4 |
| AZACITIDINE | Proctalgia | GI | 20 | 3.67 (2.37–5.70) | 4/4 | 1 | 7.32 (1.03–52.07) | 1/4 |
| AZACITIDINE | Inguinal Hernia | GI | 13 | 2.37 (1.38–4.09) | 3/4 | 1 | 12.99 (1.82–92.55) | 1/4 |
| AZACITIDINE | Incarcerated Inguinal Hernia | GI | 3 | 27.12 (8.66–84.92) | 4/4 | 1 | 591.16 (75.58–4624.20) | 2/4 |
| AZACITIDINE | Vomiting | GI | 456 | 1.13 (1.03–1.24) | 2/4 | 5 | 0.87 (0.36–2.10) | 0/4 |
| AZACITIDINE | Constipation | GI | 403 | 2.18 (1.98–2.41) | 3/4 | 1 | 0.46 (0.06–3.26) | 0/4 |
| AZACITIDINE | Gastrointestinal Haemorrhage | GI | 151 | 1.86 (1.58–2.18) | 2/4 | 2 | 2.58 (0.64–10.36) | 0/4 |
| AZACITIDINE | Stomatitis | GI | 67 | 1.27 (1.00–1.61) | 0/4 | 2 | 3.67 (0.92–14.72) | 1/4 |
| AZACITIDINE | Oral Pain | GI | 9 | 0.45 (0.23–0.86) | 0/4 | 1 | 4.64 (0.65–33.03) | 1/4 |
| AZACITIDINE | Gastrointestinal Wall Thickening | GI | 3 | 2.31 (0.75–7.18) | 0/4 | 1 | 45.47 (6.35–325.71) | 2/4 |
| AZACITIDINE | Tumour Lysis Syndrome | Met/Nut | 165 | 22.89 (19.62–26.70) | 4/4 | 1 | 33.78 (4.73–241.48) | 2/4 |
| AZACITIDINE | Hypokalaemia | Met/Nut | 92 | 2.36 (1.93–2.90) | 3/4 | 2 | 8.23 (2.05–33.01) | 2/4 |
| AZACITIDINE | Failure To Thrive | Met/Nut | 14 | 3.11 (1.84–5.26) | 3/4 | 1 | 36.49 (5.10–260.98) | 2/4 |
| AZACITIDINE | Hyponatraemia | Met/Nut | 119 | 2.44 (2.04–2.93) | 4/4 | 1 | 2.83 (0.40–20.15) | 0/4 |
| AZACITIDINE | Metabolic Acidosis | Met/Nut | 25 | 0.93 (0.63–1.38) | 0/4 | 2 | 15.39 (3.84–61.78) | 2/4 |
| AZACITIDINE | Gout | Met/Nut | 16 | 1.01 (0.62–1.65) | 0/4 | 1 | 4.33 (0.61–30.82) | 1/4 |
| DECITABINE | Constipation | GI | 116 | 1.94 (1.62–2.33) | 2/4 | 64 | 4.31 (3.37–5.52) | 4/4 |
| DECITABINE | Stomatitis | GI | 35 | 2.05 (1.47–2.86) | 3/4 | 23 | 6.14 (4.07–9.27) | 4/4 |
| DECITABINE | Gingival Bleeding | GI | 14 | 3.66 (2.17–6.18) | 4/4 | 7 | 9.26 (4.39–19.52) | 4/4 |
| DECITABINE | Haemorrhoidal Haemorrhage | GI | 4 | 2.68 (1.01–7.15) | 2/4 | 3 | 7.11 (2.28–22.15) | 4/4 |
| DECITABINE | Colitis | GI | 62 | 6.00 (4.67–7.70) | 4/4 | 5 | 1.54 (0.64–3.71) | 0/4 |
| DECITABINE | Gastrointestinal Haemorrhage | GI | 62 | 2.36 (1.84–3.03) | 3/4 | 2 | 0.36 (0.09–1.46) | 0/4 |
| DECITABINE | Proctalgia | GI | 15 | 8.53 (5.14–14.17) | 4/4 | 1 | 1.09 (0.15–7.77) | 0/4 |
| DECITABINE | Haematemesis | GI | 14 | 1.95 (1.16–3.30) | 2/4 | 2 | 1.07 (0.27–4.28) | 0/4 |
| DECITABINE | Neutropenic Colitis | GI | 14 | 26.05 (15.40–44.05) | 4/4 | 0 |  | 0/4 |
| DECITABINE | Haemorrhoids | GI | 13 | 2.39 (1.38–4.11) | 3/4 | 2 | 1.08 (0.27–4.30) | 0/4 |
| DECITABINE | Melaena | GI | 12 | 1.89 (1.07–3.33) | 2/4 | 1 | 0.59 (0.08–4.20) | 0/4 |
| DECITABINE | Ileus | GI | 11 | 3.28 (1.81–5.92) | 3/4 | 0 |  | 0/4 |
| DECITABINE | Diverticular Perforation | GI | 7 | 11.71 (5.57–24.58) | 4/4 | 0 |  | 0/4 |
| DECITABINE | Enterocolitis | GI | 7 | 4.56 (2.17–9.57) | 4/4 | 0 |  | 0/4 |
| DECITABINE | Duodenal Ulcer | GI | 6 | 3.07 (1.38–6.84) | 3/4 | 0 |  | 0/4 |
| DECITABINE | Gastrointestinal Toxicity | GI | 5 | 4.04 (1.68–9.70) | 3/4 | 0 |  | 0/4 |
| DECITABINE | Mouth Haemorrhage | GI | 5 | 2.42 (1.01–5.81) | 2/4 | 1 | 3.10 (0.43–22.09) | 0/4 |
| DECITABINE | Oesophageal Ulcer | GI | 4 | 3.79 (1.42–10.09) | 2/4 | 0 |  | 0/4 |
| DECITABINE | Rectal Fissure | GI | 4 | 22.35 (8.37–59.69) | 4/4 | 0 |  | 0/4 |
| DECITABINE | Gastrointestinal Oedema | GI | 3 | 6.05 (1.95–18.78) | 2/4 | 0 |  | 0/4 |
| DECITABINE | Anal Ulcer | GI | 2 | 9.26 (2.31–37.08) | 1/4 | 0 |  | 0/4 |
| DECITABINE | Erosive Duodenitis | GI | 2 | 8.57 (2.14–34.30) | 1/4 | 0 |  | 0/4 |
| DECITABINE | Gastrointestinal Polyp Haemorrhage | GI | 2 | 9.94 (2.48–39.79) | 1/4 | 0 |  | 0/4 |
| DECITABINE | Anal Dilatation | GI | 1 | 158.64 (21.63–1163.41) | 1/4 | 0 |  | 0/4 |
| DECITABINE | Diverticular Fistula | GI | 1 | 89.80 (12.42–649.38) | 1/4 | 0 |  | 0/4 |
| DECITABINE | Gingival Cyst | GI | 1 | 43.27 (6.04–309.92) | 1/4 | 0 |  | 0/4 |
| DECITABINE | Tooth Resorption | GI | 1 | 17.63 (2.47–125.60) | 1/4 | 0 |  | 0/4 |
| DECITABINE | Oral Pain | GI | 11 | 1.70 (0.94–3.07) | 0/4 | 7 | 4.70 (2.23–9.88) | 4/4 |
| DECITABINE | Gastrointestinal Ulcer | GI | 2 | 3.16 (0.79–12.63) | 0/4 | 1 | 5.17 (0.72–36.95) | 1/4 |
| DECITABINE | Bile Acid Malabsorption | GI | 1 | 14.29 (2.01–101.77) | 0/4 | 1 | 31.23 (4.24–229.87) | 2/4 |
| DECITABINE | Discoloured Vomit | GI | 1 | 3.90 (0.55–27.70) | 0/4 | 1 | 10.41 (1.45–74.81) | 1/4 |
| DECITABINE | Faecaloma | GI | 1 | 0.68 (0.10–4.80) | 0/4 | 1 | 4.03 (0.57–28.78) | 1/4 |
| DECITABINE | Gastric Mucosal Lesion | GI | 1 | 4.98 (0.70–35.42) | 0/4 | 1 | 22.79 (3.13–166.13) | 2/4 |
| DECITABINE | Gastric Polyps | GI | 1 | 1.53 (0.21–10.84) | 0/4 | 1 | 6.91 (0.97–49.47) | 1/4 |
| DECITABINE | Gingival Swelling | GI | 1 | 0.81 (0.11–5.74) | 0/4 | 2 | 5.94 (1.48–23.87) | 1/4 |
| DECITABINE | Loose Tooth | GI | 1 | 1.09 (0.15–7.77) | 0/4 | 1 | 6.39 (0.89–45.70) | 1/4 |
| DECITABINE | Malocclusion | GI | 1 | 13.07 (1.84–93.08) | 0/4 | 1 | 105.40 (13.18–842.90) | 2/4 |
| DECITABINE | Oral Mucosal Blistering | GI | 1 | 0.52 (0.07–3.67) | 0/4 | 1 | 3.40 (0.48–24.24) | 1/4 |
| DECITABINE | Oral Mucosal Exfoliation | GI | 1 | 2.28 (0.32–16.17) | 0/4 | 1 | 7.53 (1.05–53.93) | 1/4 |
| DECITABINE | Teeth Brittle | GI | 1 | 3.30 (0.47–23.48) | 0/4 | 1 | 19.16 (2.64–139.12) | 2/4 |
| DECITABINE | Tongue Coated | GI | 1 | 2.14 (0.30–15.19) | 0/4 | 2 | 8.65 (2.15–34.84) | 2/4 |
| DECITABINE | Tongue Erythema | GI | 1 | 4.39 (0.62–31.23) | 0/4 | 1 | 8.88 (1.24–63.68) | 1/4 |
| DECITABINE | Gingival Discomfort | GI |  |  | 0/4 | 1 | 15.61 (2.16–112.90) | 2/4 |
| DECITABINE | Decreased Appetite | Met/Nut | 96 | 1.41 (1.15–1.72) | 2/4 | 31 | 1.71 (1.20–2.43) | 1/4 |
| DECITABINE | Tumour Lysis Syndrome | Met/Nut | 47 | 19.97 (14.99–26.61) | 4/4 | 3 | 14.14 (4.52–44.26) | 4/4 |
| DECITABINE | Iron Overload | Met/Nut | 3 | 9.62 (3.10–29.85) | 4/4 | 3 | 46.86 (14.65–149.93) | 4/4 |
| DECITABINE | Eating Disorder Symptom | Met/Nut | 2 | 15.48 (3.86–62.03) | 1/4 | 2 | 33.07 (8.05–135.89) | 2/4 |
| DECITABINE | Hypoalbuminaemia | Met/Nut | 18 | 8.89 (5.60–14.12) | 4/4 | 0 |  | 0/4 |
| DECITABINE | Hypophosphataemia | Met/Nut | 16 | 8.02 (4.91–13.10) | 4/4 | 0 |  | 0/4 |
| DECITABINE | Hypervolaemia | Met/Nut | 15 | 2.47 (1.49–4.09) | 3/4 | 0 |  | 0/4 |
| DECITABINE | Failure to Thrive | Met/Nut | 12 | 8.27 (4.69–14.57) | 4/4 | 0 |  | 0/4 |
| DECITABINE | Gout | Met/Nut | 11 | 2.15 (1.19–3.88) | 3/4 | 1 | 0.63 (0.09–4.44) | 0/4 |
| DECITABINE | Hyperuricaemia | Met/Nut | 10 | 9.06 (4.87–16.85) | 4/4 | 0 |  | 0/4 |
| DECITABINE | Acidosis | Met/Nut | 8 | 3.82 (1.91–7.64) | 3/4 | 0 |  | 0/4 |
| DECITABINE | Lipomatosis | Met/Nut | 2 | 38.70 (9.62–155.61) | 1/4 | 0 |  | 0/4 |
| DECITABINE | Cell-Mediated Cytotoxicity | Met/Nut | 1 | 68.98 (9.58–496.68) | 1/4 | 0 |  | 0/4 |
| DECITABINE | Food Aversion | Met/Nut | 1 | 2.36 (0.33–16.74) | 0/4 | 1 | 7.46 (1.04–53.44) | 1/4 |
| DECITABINE | Food Refusal | Met/Nut | 1 | 4.61 (0.65–32.77) | 0/4 | 1 | 18.33 (2.53–132.95) | 2/4 |

Footnote: Abbreviations: FAERS, FDA Adverse Event Reporting System; CVARDD, Canada Vigilance Adverse Reaction Database; SOC, system organ class; GI, gastrointestinal disorders; Met/Nut, metabolism and nutrition disorders; PT, preferred term; ROR, reporting odds ratio; CI, confidence interval; PRR, proportional reporting ratio; MGPS, multi-item gamma Poisson shrinker; BCPNN, Bayesian confidence propagation neural network; EBGM05, lower limit of 95% confidence interval of the empirical Bayes geometric mean; EBGML, lower limit of 95% confidence interval of the empirical Bayes geometric mean (CVARDD nomenclature); IC025, lower limit of 95% confidence interval of the information component; C025, lower limit of 95% confidence interval of the information component (CVARDD nomenclature).

Signal detection criteria: ROR positivity was defined as the lower limit of the 95% CI > 1 with at least 3 reported cases (a ≥ 3); PRR positivity was defined as PRR ≥ 2, χ² ≥ 4, and a ≥ 3; MGPS positivity was defined as EBGM05 (FAERS) or EBGML (CVARDD) > 2; BCPNN positivity was defined as IC025 (FAERS) or C025 (CVARDD) > 0. The signal score (e.g., 4/4) indicates the number of algorithms meeting their respective positivity criteria among the four methods tested. Concordant positive: signals positive in both FAERS and CVARDD; FAERS positive / CVARDD negative: signals positive in FAERS but not meeting positivity criteria in CVARDD; CVARDD positive / FAERS negative: signals positive in CVARDD but not meeting positivity criteria in FAERS.
